# The adoption of hypertension reference framework: An investigation among primary care physicians of Hong Kong

**DOI:** 10.1371/journal.pone.0205529

**Published:** 2018-10-09

**Authors:** Yuan Fang, Harry H. X. Wang, Miaoyin Liang, Ming Sze Yeung, Colette Leung, Chun Hei Chan, Wilson Cheung, Jason L. W. Huang, Junjie Huang, Regina W. S. Sit, Samuel Y. S. Wong, Martin C. S. Wong

**Affiliations:** 1 School of Public Health and Primary Care, Chinese University of Hong Kong, Shatin, Hong Kong, Hong Kong Special Administrative Region, China; 2 School of Public Health, Sun Yat-Sen University, Guangzhou, People's Republic of China; 3 General Practice and Primary Care, Institute of Health and Wellbeing, University of Glasgow, Glasgow, United Kingdom; Medical University Graz, AUSTRIA

## Abstract

**Background:**

The Hong Kong Government released a Reference Framework (RF-HT) for Hypertension Care for Adults in Primary Care Settings since 2010. No studies have evaluated its adoption by primary care physicians (PCPs) since its release.

**Aim:**

We aimed to evaluate the level of PCPs’ adoption of the RF-HT and the potential barriers of its use in family practice.

**Design and setting:**

A cross-sectional study was conducted by a self-administered validated survey among all PCPs in Hong Kong through various means.

**Methods:**

We assessed the level of and factors associated with its adoption by multivariate logistic regression modelling.

**Result:**

A total of 3,857 invitation episodes were sent to 2,297 PCPs in 2014–2015. We received 383 completed questionnaires. The average score of adoption was 3.43 out of 4.00, and 47.5% of PCPs highly adopted RF-HT in their daily consultations. Male practitioners (adjusted odds ratio [aOR] = 0.524, 95% CI = 0.290–0.948, *p* = 0.033) and PCPs of public sector (aOR = 0.524, 95% CI = 0.292–0.940, *p* = 0.030) were significantly less likely to adopt the RF-HT. PCPs with higher training completion or being academic fellow are more likely to adopt RF-HT than those who were “nil to basic training completion” (aOR = 0.479, 95% CI = 0.269–0.853, *p* = 0.012) or “higher trainee” (aOR = 0.302, 95% CI = 0.093–0.979, *p* = 0.046). Three most-supported suggestions on RF-HT improvement were simplification of RF-HT, provision of pocket version and promoting in patients.

**Conclusion:**

Among PCP respondents, the adoption level of the RF-HT was high. These findings also highlighted some factors associated with its adoption that could inform targeted interventions for enhancing its use in clinical practice.

## Introduction

Worldwide, hypertension accounts for 7.6 million premature deaths and 92 million disability-adjusted life years (DALY) annually, and contributes to 47% of ischemic heart disease and 54% of stroke [1]. Although its prevalence is growing [2, 3], its control rate remains low in many countries [3–6]. In Hong Kong, the proportion of people diagnosed with hypertension increased from 9.3% in 2008, to 10.3% in 2009/10, and 11.0% in 2011/12 [7]. However, according to a recent territory-wide cohort study in Hong Kong [8], only 46% of respondents with high blood pressure received diagnosis. The treatment rate and control rate were only 70% and 42% among those diagnosed [8].

Primary Care Physicians (PCPs) play a crucial role to offer comprehensive, coordinated, first-contact and continuing care for hypertensive patients in the community. However, suboptimal blood pressure control is often associated with poor adherence to hypertension frameworks or guidelines among PCPs [9, 10]. Studies reported that non-adherence to the guidelines would lead to wrong posture or positioning of patients while blood pressure was taken[11, 12], omission of cardiovascular disease (CVD) risk estimation [9, 13–15], under or over treatment [6, 14, 15], poor documentation [16] and lack of recommendations on lifestyle modification provided to patients [6, 16, 17]. PCPs’ adherence to hypertension guidelines was studied in many countries but not in Hong Kong.

Barriers of adherence to guidelines among PCPs included: (a) insufficient knowledge of the guidelines [11, 18, 19] or the most updated evidence [6, 12–15, 20, 21]; (b) considered such guidelines to be not clear, outdated, not applicable or not credible [6, 11, 15, 18, 19, 22, 23]; (c) being junior PCPs [6, 11]; (f) lack of expectations on the impact of adopting guidelines [11, 18, 19, 21–26].

In 2010, the Primary Care Office of the Hong Kong Government produced the first reference framework for Hypertension Care (RF-HT) for Adults in Primary Care Settings and updated in 2013 [27] (S1 File). The reference framework was the first standardized local hypertension guidelines made for the whole healthcare system in Hong Kong, providing general references for practice in primary care settings to support the policy of promoting primary care. It displayed a different situation compared to other countries to develop realistic frameworks suitable for local culture. Its implementation in primary care is expected to provide better healthcare for hypertensive patients. However, the awareness and adoption level of this RF-HT in local PCPs has not been evaluated.

The objective of this study is to investigate the awareness, adoption level, enabling factors and barriers to the use of RF-HT among PCPs, as well as the factors associated with its adoption. We aim to analyze these parameters in the context of the well-recognized “guideline implementability framework” [28].

## Method

### Survey instrument

Based on literature review and focus group, the survey items were tailored-made to the local context of primary healthcare by a panel consisted of family medicine specialists, public health professionals and epidemiologists. A pilot-test was conducted in ten local PCPs, whereas the questionnaire was finalized based on their feedback. The adoption level of the RF-HT was assessed by PCPs’ reporting on their common practice for hypertension care, using a Likert Scale of “1 = never”, “2 = sometimes”, “3 = often”, “4 = always” which produced a score of RF-HT adoption.

### Sampling frame and methodology

The targeted population of this study was all doctors working in primary care settings whose clinical duties involved management of hypertensive patients. There were approximately 5,700 and 5,884 PCPs working in the private and public sector, between the year of 2014–2015 [29, 30]. The public sector included General Out-Patient Clinics (GOPCs) and Family Medicine Specialist Clinics (FMSCs), Specialist Out-Patient Clinics (SOPCs) and staff clinics under the jurisdiction of the Hospital Authority. Moreover, we also used a “central private practitioner registry” which was established by us and reported in our previous work [31]. After omission of duplication, it is consisted of comprehensive contact information of 2,297 registered private doctors.

As the adoption rate of guidelines reported in the literatures [11, 12, 16, 17, 19, 24, 32] ranges from 10–50%, we assumed 30% of PCPs would report that they “often” or “always” had adopted the framework. The cut-off values were chosen arbitrary for there was no agreed guideline on this. Targeting at a precision level of 0.05, a minimum of 323 subjects were required based on the standard formula (i.e. precision = 1.96×√[(p(1-p)/N)], where p is the proportion of PCPs who would highly adopt the reference framework). Given a response rate of 11.4% from our previous PCP survey in the primary care sector with no incentives [31, 33], we sent our survey invitations to all the public and private PCPs in the sampling frame in order to achieve adequate sample size to assess the representativeness. And particularly in this study, survey invitations were also sent by on-site visits and disseminated in Continuous Medical Education (CME) lunch seminars. According to our previous experience, a significantly higher response rate could be achieved by reaching more potentially eligible PCPs not included in the invitation list [31].

Hard copies of surveys were sent to the primary care clinics in the public sector via the arrangements of the respective Chief of Service (COS), who participated in the coordination of survey dissemination and collection. The surveys were all self-administered, and the completed surveys were returned via collection of the hard copies through postal means. For invitations of the PCPs in the private sector, multiple channels were used including postage, e-mail addresses and fax-lines, whichever was available. Original dissemination of e-survey was conducted in early March 2016 and three separated reminders were sent in June 2016. For surveys sent by postal means, postage-paid and self-return envelopes were provided to the participants. The PCPs were reminded not to complete a survey if they have already returned the questionnaire. To enhance response rates, we sent up to three reminders to non-respondents separated by three weeks after the date of survey invitation to the PCPs’ office. Upon completion of the questionnaire, they were given a HKD 50 supermarket coupon as an incentive. To assure the confidentiality and anonymity, each doctor’s identity was replaced by a unique identifying number before data analysis.

### Statistical analyses

The SPSS 21.0 (Chicago, Illinois) was used for data entry and analysis. The adoption level of the hypertension reference framework was calculated as an overall score and presented in proportions. In order to compare the difference between PCPs with high vs. low levels of adoption, independent t-tests or Chi-square tests were performed for continuous or categorical variables, respectively. The covariates significantly associated with adoption of RF-HT were evaluated by multiple logistic regression analyses. P values <0.05 were regarded as statistically significant.

## Result

### Characteristics of participants

A total of 3,857 surveys with the individual informed consent files were sent out through various means including postage or fax-line or electronic web-based answering system (3,255), invitation in the clusters of Hospital Authority (370), on-site visit (85) and recruitment in CME luncheons in person (147). 383 surveys were returned by these means, giving a response rate of 9.9%. The demographic characteristics of participating PCPs are shown in Table 1.

**Table 1:** Participant characteristics (n = 383).

Characteristics	Number (%)
***Age***	
≤30	36 (9.4)
31–40	88 (23.0)
41–50	87 (22.7)
51–60	88 (23.0)
>60	83 (21.7)
***Gender***	
Male	246 (64.2)
Female	135 (35.2)
***Practice experience since graduation from medical school***	
<5 years	24 (6.3)
5–10 years	34 (8.9)
11–15 years	60 (15.7)
16–20 years	74 (19.3)
21–25 years	45 (11.7)
26–30 years	49 (12.8)
>30 years	96 (25.1)
***Clinical practice***	
In public sector	182 (47.5)
In private sector	201 (52.5)
*Solo*	111 (29.0)
*With partners*	64 (16.7)
*Others*	92 (24.0)
Academic tutor	91 (23.8)
General Out-Patient Clinic	190 (49.6)
Family Medicine Integrated Clinic	93 (24.3)
***Training status in Hong Kong Academy of Medicine (HKAM)***	
Nil	131 (34.2)
Basic Trainee	34 (8.9)
Completed basic training	35 (9.1)
Higher trainee	20 (5.2)
Completed higher training	19 (5.0)
Academy fellow	137 (35.8)
***Having Specialty***	175 (46.5)
Family Medicine	110 (28.7)
Pediatrics	23 (6.0)
Others	42 (11.0)

### Difference in PCPs by adoption level of reference framework for hypertension

The adoption level was analyzed with the assumption that the recommendations of reference framework have been taken up in daily routine practice of doctor participants, whereas “strongly disagree” and “disagree” were defined as low adoption while “agree” and “strongly agree” were regarded as high adoption in each recommendation. Thus, the average score of the adoption was computed as 3.43 (SD = 0.314), whereas the overall adoption was defined as high when it obtained the score ≥3.5 out of 4. Thus it was found that 47.5% of primary care physicians strongly adopted this framework in their daily consultations. As shown in Table 2, the recommendations from reference framework can be categorized in three parts, i.e. blood pressure measurement, lifestyle modification and drug treatment. It is clear that all five recommendations of lifestyle modification were highly adopted by the participants within range of 94.0–95.8%. However, >50% of doctors had no agreement on BP measurement for adult individuals every two years. Meanwhile, only 53.3% of practitioners preferred to set target BP <130/80 mmHg. In the drug treatment part, all reached high consensus except *“annual screening urine for protein/albumin in HT patients”*. Moreover in Table 3, some characteristics of participants were found as potential associated factors of RF-HT adoption.

**Table 2:** Adoption level of recommendations in daily consultations of physicians (n = 383).

Recommendations	Proportion of participants who highly adopt the recommendation (%)
a. Measure blood pressure (BP) for hypertensive patients at every visit.	366 (95.6)
b. Measure blood pressure (BP) for high risk individuals at every visit	345 (90.1)
c. Measure blood pressure (BP) for individuals aged >18 years old, every 2 years	168 (43.9)
d. Set target BP < 130/80 mm Hg for hypertensive patients with diabetes or chronic kidney disease.	204 (53.3)
e. Consider BP <140/80 mm Hg as the optimal treatment goal for simple hypertensive patients.	343 (89.6)
f. Advice overweight/obese individuals to achieve healthy body weight.	365 (95.3)
g. Advise hypertensive patients to maintain optimal body weight and adopt healthy eating habit.	367 (95.8)
h. Advise hypertensive patients to increase regular level of physical activity.	366 (95.6)
i. Advise hypertensive patients to stop smoking, and start smoking cessation counseling.	365 (95.3)
j. Advise hypertensive patients to restrict salt intake.	360 (94.0)
k. Start drug treatment within a month If BP between 160-179/100-109 mmHg	365 (95.3)
l. Prescribe ACEI, calcium channel blocker or thiazide-type diuretic to treat hypertension.	357 (93.2)
m. Increase dosage or adding third drug from different class if BP goal was not reached after primary treatment in hypertensive patients.	364 (95.0)
n. Annual screening for Urine for protein/ albumin in hypertensive patients	316 (82.5)
o. Annual screening for Fasting blood glucose in hypertensive patients	355 (92.7)

**Table 3:** Participant characteristics according to the level of adoption of the reference framework.

Characteristics	High adoption [n = 182] (%)	Low adoption [n = 201] (%)	*p*
***Age***			
≤30	11 (6.0)	25 (12.4)	0.038*
31–60	124 (68.1)	139 (69.2)	
>60	47 (25.8)	37 (18.4)	
***Gender***			
Male	108 (59.3)	138 (56.1)	0.078
***Practice experience since graduation from medical school***			
<10 years	18 (9.9)	40 (19.9)	0.007*
>10 years	164 (90.1)	161 (80.1)	
***Clinical practice***			
Public sector	78 (42.9)	104 (57.1)	0.082
Private sector	104 (57.1)	97 (48.3)	
***Academic fellow***			
Yes	53 (29.1)	38 (18.9)	0.010*
***Having specialty***			
Yes	94 (51.6)	81 (40.3)	0.012*
***Specialty in family medicine***			
Yes	54 (48.2)	58 (51.8)	0.861
***Training status in HKAM***			
Nil to completed basic training	85 (48.2)	115 (57.5)	0.008*
Higher trainee	5 (2.8)	15 (7.5)	
Completed higher training to academic fellow	86 (48.9)	70 (35.0)	

The Chi-square test was employed for the analysis of categorical data.

*P-value <0.05 is considered as at significant level.

**Fig 1:**
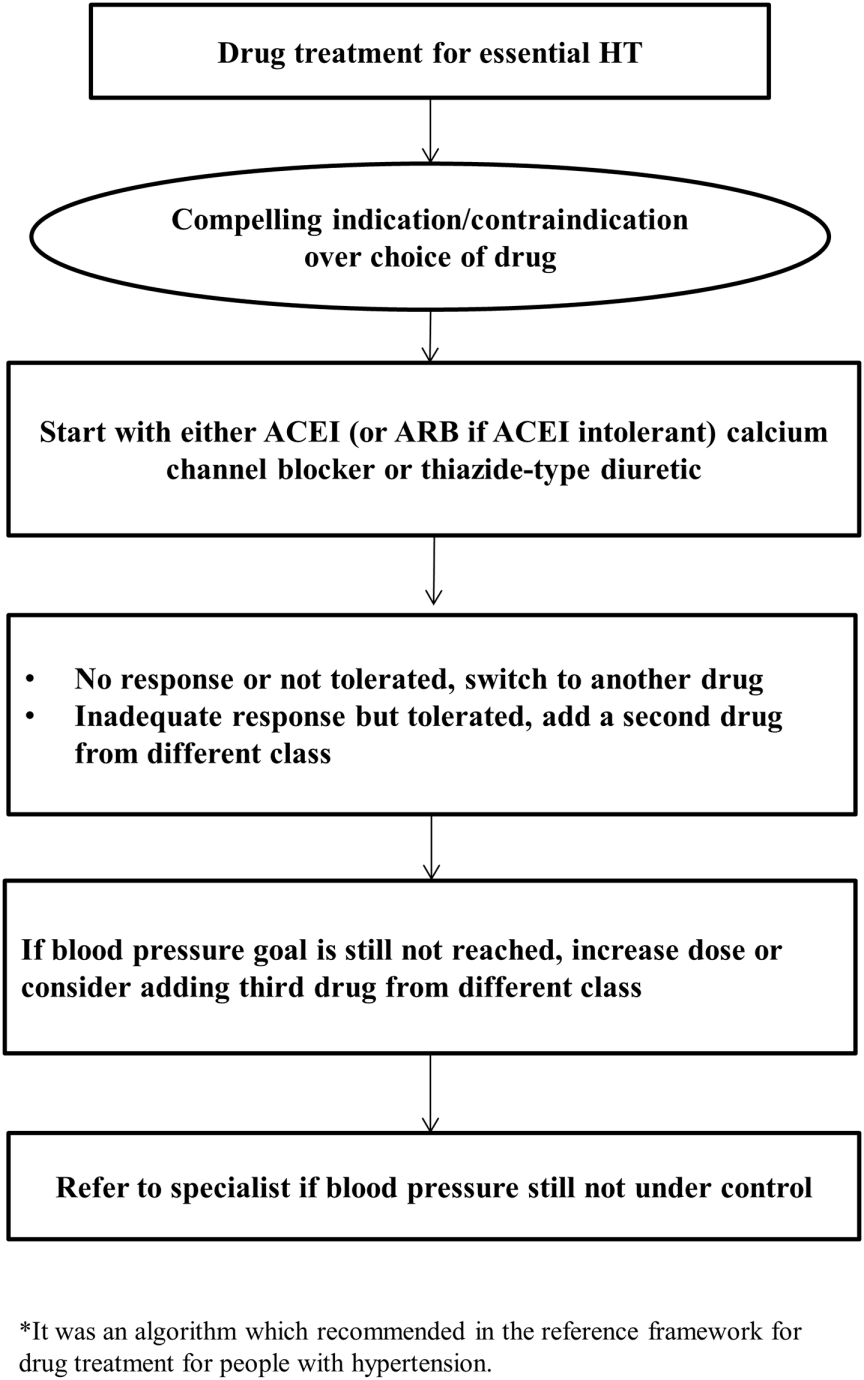
Treatment algorithm for pharmacological management of hypertension.

**Fig 2:**
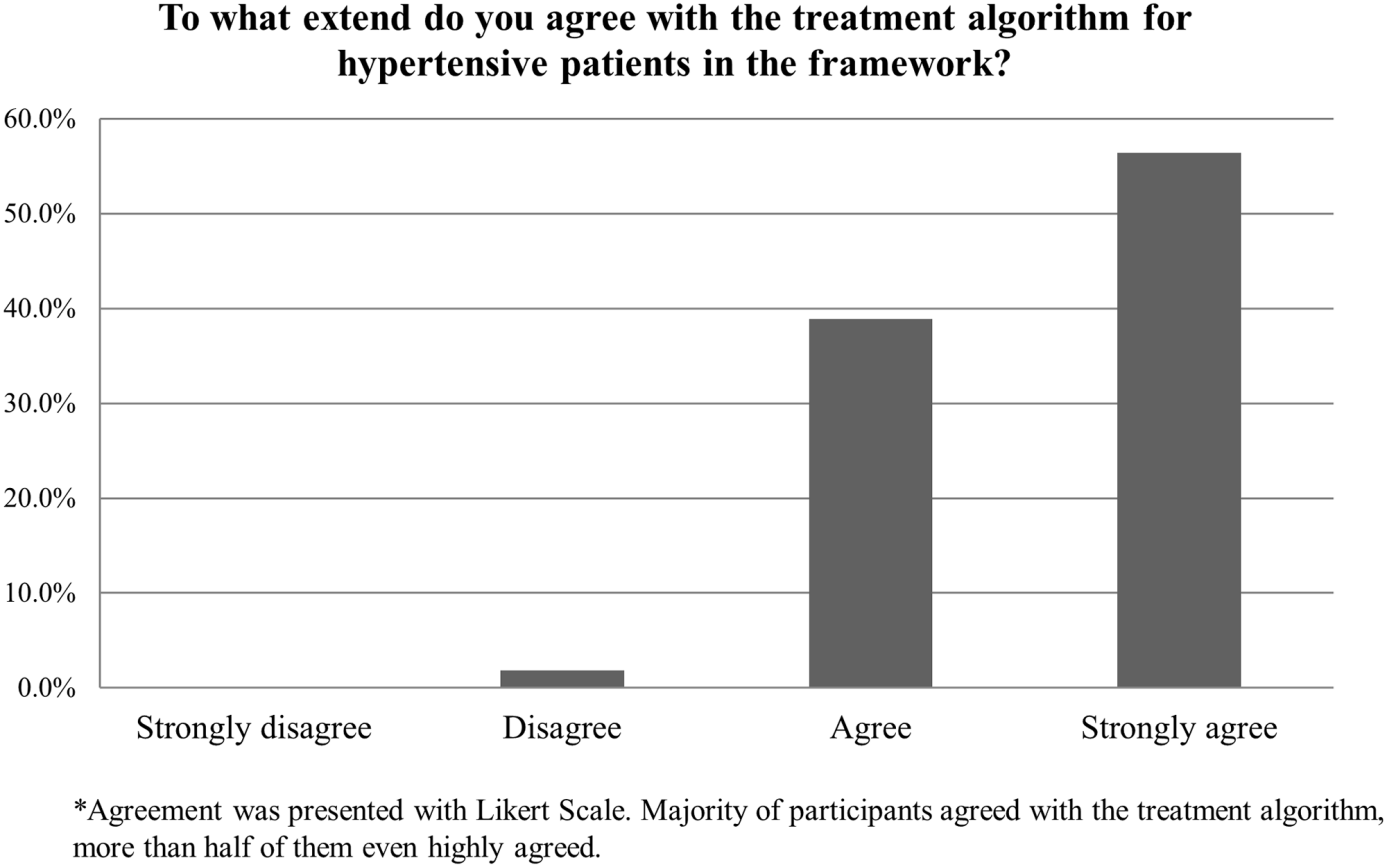
The extend of agreement with the treatment algorithm for hypertensive patients.

In addition, Fig 1 showed the treatment algorithm for hypertensive patients which was recommended in the reference framework. The majority of participants highly agreed with the treatment algorithm (Fig 2). Moreover, 371 out of 383 physicians highly adopted the reference framework to initiate drug treatment when patient’s blood pressure is greater than 160 mmHg after undergoing lifestyle modification (S1 Table). However, a total of 64.7% of the participants showed disagreement to initiate the drug treatment with thiazide-type diuretic as the first line drug treatment; instead, angiotensin-converting-enzyme inhibitor (ACEI) or calcium channel blocker (CCB) was mostly considered by health professionals (data not shown).

### Factors related to the adoption of reference framework

Potential factors associated with reference framework adoption were divided to four themes, including guideline-related, patient-related, PCP-related and external factors. S2 Table presented potential factors affecting adoption at different levels. Multivariate logistic regression was used to evaluate factors significantly associated with the adoption level of the framework (shown in Table 4). The results showed that 1) male PCPs were less likely to adopt RF-HT than female PCPs (adjusted odds ratio [aOR] = 0.524, 95%CI = 0.290–0.948, *p* = 0.033); 2) PCPs who worked in public sector were less likely to adopt RF-HT (aOR = 0.524, 95%CI = 0.292–0.940, *p* = 0.030); 3) PCPs whose training status in Hong Kong Academy of Medicine (HKAM) were “Nil to completed basic training” (aOR = 0.479, 95%CI = 0.269–0.853, *p* = 0.012) or “Higher trainee” (aOR = 0.302, 95%CI = 0.093–0.979, *p* = 0.046) were less likely to have high RF-HT adoption, and 4) being academic fellow was a marginal significant factor for RF-HT adoption (aOR = 1.748, 95%CI = 0.982–3.111, *p* = 0.058).

**Table 4:** Factors associated with adoption of the reference framework by multivariate logistic regression analysis.

	Adjusted Odds Ratio	95% CI		*p*
		Lower	Upper	
***Gender***				
Female	**Reference**			
Male	0.524	0.290	0.948	0.033*
***Clinical practice***				
Private sector	**Reference**			
Public sector	0.524	0.292	0.940	0.030*
***Training status in HKAM***				
Completed higher training to Academic fellow	**Reference**			
Higher trainee	0.302	0.093	0.979	0.046*
Nil to Completed basic training	0.479	0.269	0.853	0.012*
***Academic tutor***				
No	**Reference**			
Yes	1.748	0.982	3.111	0.058

*P-value <0.05 is considered as at significant level.

Furthermore, suggestions to further improve the adoption level of reference framework among doctors were shown in Table 5, i.e. simplification of RF-HT (91.9%), provision of pocket versions (86.4%) and RF-HT promotion in patients in a patient-centered manner (81.9%).

**Table 5:** Adoption level of improvements on enhancing the use of reference framework for hypertension care in your clinical practice (n = 383).

Suggestions on improvement	Proportion of participants who highly adopt the suggestions (%)
a. Simplifying the framework into flow sheets or slogans.	351 (91.9)
b. Providing pocket versions (i.e. mobile phone apps).	330 (86.4)
c. Providing multi-lingual patient version.	301 (79.0)
d. Including a referral system, with contact information of other medical care providers.	289 (75.9)
e. Scheduling the implementation process of primary care physicians.	282 (74.4)
f. Promoting framework to patients.	313 (81.9)
g. Easing financial burden on patients through medical fee waiving mechanism.	280 (74.1)
h. Monitoring the compliance of the reference framework.	295 (77.4)

## Discussion

### Summary

In the current study, the adoption level, as well as associated factors of PCPs with respect to the reference framework for hypertension in Hong Kong was evaluated and reported. The average score of adoption was high as 3.43/4.00, and 47.5% of PCPs highly adopted this framework in their daily consultations. Gender, practice sector and training status were indicated as significant associated factors to adoption level by multivariate logistic regression. The most suggested modifications of the guidelines are simplification of RF-HT, provision of pocket version and promoting in patients.

### Strengths and limitations

Since there was no standardized local hypertension guideline or recommendation applicable for the whole healthcare system in Hong Kong before 2010, RF-HT developed by Department of Health, HKSAR is the first-ever standardized recommendations of hypertension care covering the whole system. Hence, it is desirable and necessary to evaluate the adoption level and associated factors regarding this first-ever standardized RF-HT. This evaluation shall deliver important messages for further improvement and updates.

There are several strengths apparently observed in this study. Firstly, we recruited participant PCPs who were working in both the public and private sectors, allowing us to collect a wide variety of opinions. Secondly, the targeting results were obtained by a validated survey, which helped to clarify the situation of RF-HT adopted by PCPs in Hong Kong. Thirdly, findings of this study showed that the RF-HT was highly adopted among the PCPs in Hong Kong with associated factors identified. These strengths may provide stakeholders and policy makers with a clear blueprint for further improvement of the RF-HT and also the future development of other local guideline and reference.

In the meantime, the study had some limitations. We contacted and sent invitations to primary care practitioners in Hong Kong based on contact information from several databases. However, participants who were eligible may not be reached, and this may reduce the generalizability of the findings. The low response rate (9.9%) may affect its representativeness. Moreover, response bias may exist in self-report, and recall bias may be inevitable when asking PCPs on the details in their practices. Therefore, the high adoption rate of the RF-HT could be coincidental. Nevertheless, the findings that recommendations in the RF-HT were integrated into their regular hypertensive management implied high acceptance of the reference framework among PCPs and the capability of translating recommendations into practice.

### Comparison with existing literature

In contrast to the moderate or low adherence of hypertension guidelines reported in other countries, this study showed a high adoption of the RF-HT in Hong Kong. Significant differences in reference framework adoption were observed associated with the demographic characteristics. The proportion of participants who reported older age, longer practice experience after graduation, higher level of training status, having medical specialty and working as academic tutor, were more likely to adopt the reference framework. In existing literatures, age and practice experience are two frequently-discussed factors of PCP adherence to guidelines. Our findings are consistent with that reported by the literature [34], i.e. older PCPs who have longer practice experience may stick to the guidelines, and are less affected by external circumstances.

Moreover, most of the recommendations given on BP measurement, lifestyle modification and drug treatment were highly-adopted by PCPs except three recommendations with relatively low adoption. These implied that (a) lifestyle modification should have been widely recommended to patients in clinical practice, which showed a much higher adoption by PCPs in Hong Kong than other regions [10, 16, 17]; (b) there is no consensus on “*Set target BP < 130/80 mm Hg”*, which may be also a clinical issue in Hong Kong. This may indicate that some physicians may lack incentives in achieving BP target [10]. (c) The moderate adoption of annual uric acid screening for hypertensive patients may be due to the fact that thiazide-type diuretic was not considered as a first line drug for hypertension by 64.7% of PCPs.

Furthermore, potential associated factors indicated by multivariate logistic regression consisted of both demographic factors and independent factors identified from four themes of potential factors of RF-HT adoption in this study. The results suggest that a) PCPs’ postgraduate education may play an important role in guideline adherence as reported in the literature; b) PCPs with higher qualifications may have more accessibility to RF-HT. One potential explanation is that this may be related to their teaching responsibility during their vocational training because they may have teaching duty during their academic work.

Several effective solutions were reported in the literatures to improve the adherence rate of reference framework or guideline for chronic disease management, these include establishment of a professional team formed by physicians, pharmacists, nurses and related staff [18, 35], electronic documentation and computer-aided decision making system [16, 18, 21, 36, 37], reminder delivery to PCPs [38] and promotion of continuing medical education (CME) in PCPs [6, 12, 18, 20, 21, 39]. In this study, simplifying RF-HTs is strongly supported by PCPs in order to make it easier to understand and make it more accessible. Specifically, provision of pocket version is one of suggested vehicles to increase RF-HT adoption, e.g. mobile apps. At the same time, most of participants expected that RF-HT should be promoted to patients, which they believed could improve patients’ knowledge of hypertension and motivate them to adopt healthier lifestyles.

### Implications for research and/or practice

There was high adoption to the recommendations in RF-HT among PCPs in Hong Kong in this cross-sectional survey. Factors associated with adoption to reference framework were identified. Future work can make use of these findings to improve and reinforce the implementation of this framework. Moreover, making the RF more accessible and portable may help to further improve the adoption rate in this population.

## Ethical approval

The study protocol conformed to the ethical guidelines of the Declaration of Helsinki. This study was approved by the Survey and Behavioural Research Ethics Committee of the Chinese University of Hong Kong.

## Supporting information

S1 File Hong Kong reference framework for hypertension care for adults in primary care settings (2013). (PDF)

S1 Table Potential factors influencing the adoption of the reference framework. (DOCX)
