# Supplementary material for: The adoption of hypertension reference framework: An investigation among primary care physicians of Hong Kong
Source: PLoS One. 2018 Oct 9;13(10):e0205529. doi: 10.1371/journal.pone.0205529 (PMC6177174; doi:10.1371/journal.pone.0205529)
Supplement: S1 File — (PDF) [file pone.0205529.s001.pdf]

Hong Kong Reference Framework for Hypertension Care for Adults in Primary Care Settings

2013

Developed by: 基層醫療概念模式及預防工作常規專責小組 Task Force on Conceptual Model and Preventive Protocols, 基層醫療工作小組 Working Group on Primary Care, 食物及衞生局 Food and Health Bureau

With the professional advice of: 衞生署 Department of Health; 醫院管理局 HOSPITAL AUTHORITY

Supported by:

香港醫學專科學院
Hong Kong Academy of Medicine

香港社會醫學學院
Hong Kong College of Community Medicine

香港家庭醫學學院
The Hong Kong College of Family Physicians

香港中文大學醫學院
Faculty of Medicine, The Chinese University of Hong Kong

香港大學李嘉誠醫學院
Li Ka Shing Faculty of Medicine, The University of Hong Kong

香港西醫工會
Hong Kong Doctors Union

香港醫務委員會執照醫生協會
The Association of Licentiates of Medical Council of Hong Kong

香港醫學會
The Hong Kong Medical Association

香港糖尿聯會
Diabetes Hongkong

香港心臟專科學院
Hong Kong College of Cardiology

香港內分泌學會
Hong Kong Society of Endocrinology, Metabolism and Reproduction

基層醫療 PRIMARY CARE

Content

Preface		i
Key To Evidence Statements And Grades Of Recommendations		iii
Statement Of Intent		v
Chapter 1.	Epidemiology	1
Chapter 2.	Population-based Intervention And Life Course Approach	2
Chapter 3.	Role Of Primary Care In The Management Of Hypertension	3
Chapter 4.	Patient Education	4
Chapter 5.	Aim Of The Framework	4
Chapter 6.	Component 1: Prevention Of Hypertension - Adoption Of A Healthy Lifestyle	5
Chapter 7.	Component 2: Early Identification Of People With Hypertension	6
Chapter 8.	Component 3: Clinical Care Of Adults With Hypertension	9
	8.1 Initial assessment and investigation of the hypertensive patient	9
	8.2 Treatment of adults with hypertension	10
	8.3 Target values for blood pressure lowering	15
	8.4 Follow up and monitoring	17
	8.5 Referral	18
Chapter 9.	Component 4: Patient Empowerment	20
Chapter 10.	Future Direction To Promote The Use Of The Framework	21

<u>Modules</u>

Module 1	Framework For Population Approach In The Prevention And Control Of Hypertension Across The Life Course
Module 2	Blood Pressure Measurement
Module 3	Secondary Hypertension
Module 4	Evaluation For All Newly Diagnosed Hypertensive Patients
Module 5	Dietary Intervention
Module 6	Exercise Recommendations To People With Hypertension
Module 7	Drug Treatment For People With Hypertension
Module 8	Annual Assessment

Preface

Enhancing primary care is one of the proposals put forward in the Healthcare Reform Consultation Document "Your Health, Your Life" and has received broad public support during the first stage of public consultation conducted in 2008. In recognition of the broad support for the proposals, the Working Group on Primary Care (Working Group) under the Health and Medical Development Advisory Committee chaired by the Secretary for Food and Health was reconvened to discuss and provide strategic recommendations on enhancing and developing primary care in Hong Kong.

Four Task Forces have been established to study specific proposals set out in the Healthcare Reform Consultation Document. One of them is the Task Force on Conceptual Model and Preventive Protocols (Task Force). The Task Force makes recommendations to the Working Group on conceptual models that are evidence based with associated reference frameworks for use in the local primary care settings. The Task Force is also responsible for promulgating, maintaining and revising the models and frameworks, and the strategies to promote their adoption.

After a series of discussions with stakeholders, the Task Force has developed a basic conceptual model for the management of chronic disease using a population approach across life-course. It is based on the recognition that we need a comprehensive and continuous approach to care focused on the person to meet their needs and address their risks. The reference frameworks cover primary prevention and lifestyle changes, assessment of high risk groups, early detection and management of diseases as well as ensuring the quality of care for more complicated conditions or disabilities within the community. The need to coordinate inputs from multi-disciplinary teams, engage patients and interface with the community and other sectors is also highlighted.

To date, two reference frameworks, one on diabetes and the other on hypertension, have been developed. These reference frameworks consist of a core document supplemented by a series of different modules addressing various aspects of disease management which aim to -

(a) provide a common reference to guide and co-ordinate care to patients from all healthcare professionals across different sectors in Hong Kong for the provision of continuous, comprehensive and evidence-based care for diabetes and hypertension in the community;
(b) empower patients and their carers; and
(c) raise public's awareness on the importance of preventing and properly managing these two major chronic diseases.

Drawing on international experience and best evidence, these frameworks provide general reference for practice in primary care settings to support the policy of promoting primary care within Hong Kong. However, since clinical practice and patient engagement need to keep pace with scientific advancements, in order to ensure the latest medical developments and evidence are reflected in the frameworks to provide reference for best practice, two Clinical Advisory Groups under the Task Force have been established to review and update the reference frameworks on a regular basis. The Clinical Advisory Groups are composed of experts from academia, professional organisations, private and public primary care sector and patient groups who are members of the groups in their own right, not representing organisations.

To facilitate the promulgation and adoption of the reference frameworks, support and endorsement from healthcare professionals across different sectors in Hong Kong has been and will continue to be very important. We hope that the adoption of the reference frameworks will improve patient care by facilitating co-ordination of their care, strengthen management continuity, promote evidence based effective and efficient practice, empower patients and their carers as well as enhancing public awareness about the prevention and management of these two major chronic diseases in our community.

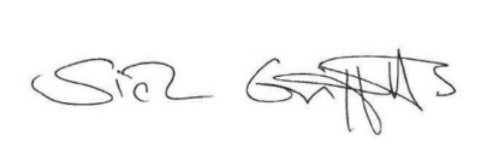

Professor Sian GRIFFITHS
Convenor
Task Force on Conceptual Model and Preventive Protocols

Key To Evidence Statements And Grades Of Recommendations

Levels of Evidence

1++	High quality meta-analyses, systematic reviews of RCTs, or RCTs with a very low risk of bias
1+	Well conducted meta-analysis, systematic reviews of RCTs, or RCTs with a low risk of bias
1-	Meta-analyses, systematic reviews of RCTs, or RCTs with a high risk of bias
2++	High quality systematic reviews of case control or cohort studies<br>High quality case control or cohort studies with a very low risk of confounding, bias, or chance and a high probability that the relationship is causal
2+	Well conducted case control or cohort studies with a low risk of confounding, bias, or chance and a moderate probability that the relationship is causal
2-	Case control or cohort studies with a high risk of confounding, bias, or chance and a significant risk that the relationship is not causal
3	Non-analytic studies, e.g. case reports, case series
4	Expert opinion

Scottish Intercollegiate Guidelines Network (SIGN) classification.

Grades of Recommendation

A	At least one meta-analysis, systematic review, or RCT rated as 1++, and directly applicable to the target population; or<br>A systematic review of RCTs or a body of evidence consisting principally of studies rated as 1+, directly applicable to the target population, and demonstrating overall consistency of results
B	A body of evidence including studies rated as 2++, directly applicable to the target population, and demonstrating overall consistency of results; or<br>Extrapolated evidence from studies rated as 1++ or 1+
C	A body of evidence including studies rated as 2+, directly applicable to the target population and demonstrating overall consistency of results; or<br>Extrapolated evidence from studies rated as 2++
D	Evidence level 3 or 4; or<br>Extrapolated evidence from studies rated as 2+

Statement Of Intent

The framework is constructed from global evidence of best practice. As with all guidance it aims to support decision making, recognising that all patients are unique and have their own needs. The Task Force endeavours to provide accurate and up-to-date information. The frameworks provide support for decision making and as such are not mandatory. They should not be construed as within any legal framework, rather as guidance for professional practice. Standards of care for individual patients are determined on the basis of all the facts and circumstances involved in a particular case. They are subject to change as scientific knowledge and technology advances and patterns of care evolve. Management of diseases must be made by the appropriate primary care practitioners responsible for clinical decisions regarding a particular treatment procedure or care plan. The responsible primary care practitioners should only arrive at a particular treatment procedure or care plan following discussion with the patient on the diagnostic and treatment choices available.

Chapter 1. Epidemiology

Hypertension means high blood pressure. Systolic blood pressure ≥ 140mm Hg or diastolic blood pressure ≥ 90mm Hg is considered high[1]. If hypertension is not well controlled and treated, it will increase the risk of cardiac failure, coronary heart disease, renal failure and stroke.

Hypertension is prevalent in Hong Kong. The Population Health Survey 2003 - 04 of the Department of Health revealed that around 27% of the population aged 15 or above had increased blood pressure[2]. The prevalence increased with advancing age, with 5.2% among those aged 15 to 24 and up to 73.3% for those aged 75 and above (Table 1). Moreover, among those 27% with increased blood pressure, more than half (15.1%) were unaware of their condition, only found to have their blood pressure raised during the survey.

Table 1: Prevalence of hypertension in Hong Kong by age groups[2]

Age Group (Years)	Self-reported, doctor diagnosed hypertension (%)	Undiagnosed but measured (%)	Total (%)
15-24	0.7	4.5	5.2
25-34	1.6	6.0	7.6
35-44	5.0	12.3	17.3
45-54	12.7	18.4	31.1
55-64	24.1	24.3	48.4
65-74	36.4	29.9	66.3
≥ 75	41.5	31.8	73.3
All age groups	12.1	15.1	27.2

Chapter 2. Population-based Intervention And Life Course Approach

Hypertension, like other chronic health conditions, poses a formidable challenge to the healthcare delivery system. Traditional care services organised to respond to acute patient problems cannot adequately serve the needs of persons with chronic diseases such as hypertension. A more systematic approach to care is required. In addition, a multidisciplinary team approach has consistently shown to be effective in achieving blood pressure control of patients, as reported in clinical trials and many practice settings. In recent years, population-based approach in the control and management of chronic diseases is emphasised. This approach seeks to embrace the whole spectrum of the problem from health promotion, disease prevention and treatment to rehabilitation. To achieve this overarching goal, a proactive approach covering primary, secondary and tertiary levels of prevention is adopted. This involves promotion of healthy behaviours to reduce disease risk, early disease detection, and quality management with the ultimate goal to reduce the incidence of complications and associated morbidities and mortality in the population.

The risks of developing chronic diseases including hypertension and cardiovascular diseases are influenced by factors acting at all stages of life. The effects of these modifiable risk factors accumulate with increasing age, especially in predisposed individuals. Major chronic diseases often share common risk factors e.g. undesirable environmental conditions, social deprivation, unhealthy dietary habit, physical inactivity, alcohol misuse and smoking. Thus, it is necessary and advantageous to adopt an integrated and life course approach in the prevention and control of chronic diseases based on the needs and risks of different population sub-groups to prevent the onset of diseases and reduce the rate of disease progression. Module 1 summarises a comprehensive approach that involves different hypertension prevention or proactive management strategies that are most relevant for the different stages of the life course.

Chapter 3. Role Of Primary Care In The Management Of Hypertension

Primary care is the first point of contact in the healthcare system and is easily accessible to the majority of the population. With support and training, primary care practitioners form an invaluable workforce in the community to deliver coordinated care to hypertensive patients, especially those with clinically stable conditions and to identify high risk subjects for referral to other experts. By applying the principles of family medicine and working in partnership with other healthcare professionals such as dietitians, nurses, occupational therapists, optometrists, pharmacists and physiotherapists, primary care practitioners are in a prime position to provide patient-centered, continuing and comprehensive care taking into account individual patients' needs and values.

In the management of chronic diseases such as hypertension, it is desirable for primary care practitioners to provide ongoing education to reduce risks, diagnose disease early, assess patients' needs, monitor treatment responses and adherence, and identify treatment barriers such as patients' concerns and misperceptions. Furthermore, they could provide holistic care by treating concurrent illnesses and co-morbidities, addressing their patients' psychosocial concerns, empowering them to change behaviour and enabling them to develop coping skills for special occasions, e.g. marriage, pregnancy, travelling and sick day management. Due to the large scope of services involved in the primary, secondary and tertiary prevention of hypertension and associated complications, multidisciplinary care targeting at interfaces between different sectors is essential. Therefore, close collaboration and coordination between primary and secondary care teams are required.

Chapter 4. Patient Education

Patient education is the cornerstone of hypertension management where patients (and their carers) are empowered with appropriate knowledge and skills to live with the disease. Patients with hypertension must be given basic knowledge about the nature, consequences and treatment of the disease as well as their rights and responsibilities in terms of access to care, adherence to recommended treatment and self-management. Primary care practitioners and other care professionals should help dispel misconceptions and address patients' concerns about the disease and its treatment, e.g. fear for long-term medication, and emphasise the positive aspects of the disease in terms of risk awareness, adoption of a healthy lifestyle and regular surveillance by a health care team[3].

Chapter 5. Aim Of The Framework

The Reference Framework for Hypertension Care in Adults in Primary Care Setting provides an updated evidence-based approach and recommends core interventions to influence current practice with a view to reducing the burden of long-term complications, including cardiovascular diseases. The Framework also aims to provide adults with or at risk of developing hypertension with a reference for better self-management and proactive disease control.

The Framework has adopted the levels of evidence and grades of recommendations proposed by the Scottish Intercollegiate Guidelines Network (SIGN). In general, grade A recommendation is supported by level 1 evidence, whilst levels 2 and 3 evidence are considered as fair evidence.

Chapter 6. Component 1: Prevention Of Hypertension Adoption Of A Healthy Lifestyle

There are two complementary approaches to reducing the incidence of hypertension in the population:

- The 'population approach', the aim of this approach is to reduce the average level of risk for developing hypertension across the entire population. The interventions required include:
  - » increasing physical activity levels,
  - » improving diet and nutrition,
  - » preventing excess dietary salt intake, and
  - » preventing and reducing overweight and obesity.
- The 'individual-based/high-risk approach', which aims to identify those at increased risk of developing hypertension, offer them appropriate advice on how to reduce the risk, and support them to lose weight and increase their physical activity levels. People with multiple risk factors for developing hypertension should also be given advice and support opportunistically to minimise their risk.

Recommendations

**Advise individuals at increased risk of developing hypertension and patients with hypertension to maintain optimal body weight, restrict dietary salt intake, abstain from smoking and practise healthy lifestyles.**	**A**

Supporting evidence

• Individuals with above-optimal blood pressure, including stage 1 hypertension (refer to Table 1), can make multiple lifestyle changes such as weight loss, sodium reduction, increased physical activity and dietary changes, to help lower their blood pressure and reduce cardiovascular risk[4].	1+

Chapter 7. Component 2: Early Identification Of People With Hypertension

Routine blood pressure measurement is of value in detecting hypertension and also provides a useful record of baseline blood pressure in normotensive patients. Classification of blood pressure and recommendations on the frequency of blood pressure screening are depicted in Table 1. For accurate blood pressure measurement, please refer to Module 2.

Recommendations

**Blood pressure measurement in all adults from 18 years of age at least every 2 years[5].**	**A**

Table 1. Classification [Note 1] of blood pressure and recommendations for frequency of blood pressure screening (Adapted from the seventh report of the Joint National Committee on Prevention, Detection, Evaluation, and Treatment of High Blood Pressure)[1]

Blood pressure classification	Initial Blood Pressure (mmHg) [Note 2, Note 4]		Recommended minimum review period [Note 3]	Action
	Systolic	Diastolic		
Normal	<120	<80	Recheck in 2 years	Encourage to adopt healthy lifestyle
Pre-hypertension	120-139	80-89	Recheck in 1 year	Lifestyle modification
Stage I hypertension	140-159	90-99	Confirm within 2 months	Lifestyle modification

Blood pressure classification	Initial Blood Pressure (mmHg) Note 2, Note 4		Recommended minimum review period Note 3	Action
	Systolic	Diastolic		
Stage II hypertension	160-179	100-109	Evaluate within one month	• Treat within one month<br>• Lifestyle modification
	>180	>110	Further evaluation within one week	• If high blood pressure is confirmed, drug treatment should be commenced<br>• May warrant urgent referral if patient presents features suggestive of malignant hypertension (Refer to Box 2)<br>• Lifestyle modification

Note 1. The classification is based on the average of 3 or more properly measured seated blood pressure readings, at least 1 week apart on office visit [6].

Note 2. If systolic and diastolic categories are different, follow recommendations for shorter review period.

Note 3. Modify review period according to reliable information about past blood pressure measurements, other cardiovascular risk factors, or target organ diseases.

Note 4. When considering a diagnosis of hypertension, measure blood pressure in both arms[7]:

- If the difference in readings between arms is more than 20 mmHg, repeat the measurements.
- If the difference in readings between arms remains more than 20 mmHg on the second measurement, measure subsequent blood pressure in the arm with the higher reading.

Chapter 8. Component 3: Clinical Care Of Adults With Hypertension

Effective treatment of hypertension can prevent or delay many of its complications, especially cardiovascular and renal complications. Medication for the control of blood pressure also plays an important role. More importantly, the management of hypertension depends on the commitment of the patient to participate proactively in self-management, adopting healthy lifestyle practices, and prompt detection and treatment of emerging cardiovascular risk factors and complications.

8.1 Initial assessment and investigation of patient with hypertension

Upon diagnosis, primary care practitioners should perform comprehensive assessment to assess co-existing cardiovascular risk factors or other problems that may affect prognosis and treatment (Box 1). Module 3 denotes the findings suspicious of secondary hypertension. Module 4 provides details regarding the assessment and investigations for individual newly diagnosed with hypertension.

Box 1 Cardiovascular risk factors[1]

- Cigarette smoking
- Obesity
- Physical inactivity
- Dyslipidaemia
- Diabetes mellitus
- Microalbuminuria or estimated GFR < 60 ml/min
- Age (older than 55 for men, 65 for women)
- Family history of essential hypertension and premature cardiovascular disease (men under 55 or women under 65)

8.2 Treatment of adults with hypertension

The aim of the treatment is to obtain maximal reduction in overall cardiovascular risk which requires:

- Correcting risk factors e.g. lifestyle modification, smoking cessation
- Maintaining good blood pressure control, and
- Monitoring potential complications and timely referral to specialist care when indicated.

8.2.1 Lifestyle modification

Adoption of a healthy lifestyle is critical for the prevention of high blood pressure and is an indispensable part of the management of those with hypertension[8]. Lifestyle modifications including healthy eating, dietary salt restriction, regular physical activity and stress management can reduce blood pressure, enhance antihypertensive drug efficacy, and reduce cardiovascular risk.

8.2.1.1 Weight control

Recommendations

**Encourage overweight and obese[a] hypertensive patients to lose weight.**	**A**

[a] World Health Organization (WHO) recommended Body Mass Index (BMI) cut-off points for overweight as 25 kg/m^2 and obesity as 30kg/m^2 in 2004 (ref. Appropriate body mass index for Asian populations and its applications for policy and intervention strategies. WHO Expert Consultations. Lancet 2004; 363: 157-63). For Asian populations, the BMI cut-off points of 23 kg/m^2 and 27.5 kg/m^2 were added as points for public health action.

Supporting evidence

- A study showed that weight loss of 9.5 lbs (4.3 kg) among overweight adults reduced systolic blood pressure by 3.7 mmHg and diastolic blood pressure by 2.7 mm Hg at six months[9]. Another study showed that an 18-month weight loss intervention program was significantly associated with a 77% long term reduction in the incidence of hypertension among subjects with blood pressure in the high normal range[10]. 1+

8.2.1.2 Healthy eating

Recommendations

**Increase consumption of fruits and vegetables to five portions per day, and reduce total and saturated fat consumption.**	**A**

Supporting evidence

- Patient with high blood pressure is benefited by adoption of the Dietary Approaches to Stop Hypertension (DASH) eating plan[11] which is a diet rich in fruits, vegetables, and low in fat dairy products with a reduced content of dietary cholesterol as well as saturated and total fat. (Module 5) 1+

8.2.1.3 Dietary sodium restriction

Recommendations

**Encourage all hypertensive patients to reduce salt intake to less than five grams (around one teaspoon of table salt) per day and not to use added salt.**	**A**

Supporting evidence

• Systolic and diastolic blood pressure could be lowered by 6.7mmHg and 3.5mmHg respectively through decreasing daily dietary salt intake from 9 grams to 3 grams among a group of adults with blood pressure exceeding 120/80mmHg[11,12,13]. (Module 5)	1+

8.2.1.4 Physical activity

Recommendations

**Advise hypertensive patients to increase level of physical activity and take regular exercises.**	**A**

Supporting evidence

• Regular aerobic exercises among hypertensive individuals were associated with 4.94mmHg reduction of systolic blood pressure and 3.73mmHg reduction of diastolic blood pressure[14]. (Module 6)	1+

8.2.1.5 Alcohol consumption

Recommendations

Recommendation	Grade
**Reduce alcohol intake in hypertensive patients to no more than two standard drinks per day[b] for men and one standard drink per day for women.**	**A**

Supporting evidence

Evidence	Level
• Alcohol reduction was associated with a significant reduction in mean systolic and diastolic blood pressure. A dose-response relationship was found between the mean percentage of alcohol consumption and mean blood pressure reduction[15].	1++
• In large population-based studies, the incidence of hypertension is increased among those who drink more than 3 drinks per day, either in a linear dose-response relationship or with a threshold wherein smaller quantities are associated with a modest decrease. Chronically, the incidence of hypertension is increased among women who drink more than two drinks per day and among men who drink more than three per day[16,17]. The cessation of heavy drinking is usually followed by significant fall in blood pressure[18].	1++

b Each standard drink contains 10 grams of pure alcohol. Defining one standard drink as 10 grams of pure alcohol, it equates to about 250 ml of regular beer at 5% of alcohol content, one small glass (100 ml) of wine at 12% alcohol content, or one pub measure (30ml) of hard liquor at 40% of alcohol content.

8.2.1.6 Stop smoking

Recommendations

**Encourage all hypertensive patients to stop smoking.**	A

Supporting evidence

• Smoking cessation is the most effective, immediate way to reduce cardiovascular risk. The pressor effect of smoking could be partly responsible for the major increase in stroke and coronary disease among smokers, as well as for the apparent resistance to antihypertensive therapy[19,20].	1++
• Smoking exerts a major pressor effect on ambulatory blood pressure monitoring[21]. The use of smokeless tobacco and cigars may also raise blood pressure[22]. Smoking cessation could reduce the overall cardiovascular risk as there was a clear dose-response relationship between the number of cigarettes smoked per day and risk of developing both coronary heart disease and stroke[23].	1++<br>2++

If assistance is needed, please refer to Appendix 1 for more information on smoking cessation services.

8.2.2 Drug treatment

Therapy begins with lifestyle modification, and if blood pressure goal is not achieved, drug treatment must often be considered in addition. The choice of drug treatment should take into account the specific indications and contraindications. (Module 7)

Recommendations

**Consider to start drug treatment in patients with sustained systolic blood pressures ≥ 140mmHg or diastolic blood pressures ≥ 90mmHg despite lifestyle modification for 6 months or if target organ damage is present.**	A

Supporting evidence

• The Hypertension Optimal Treatment (HOT) randomised trial showed that intensive lowering of blood pressure in patients with hypertension was associated with a low rate of cardiovascular events[24].	1++

8.3 Target values for blood pressure lowering

The relationship between blood pressure and cardiovascular risk is continuous. In general, the higher the risk of the individual patient, the more aggressive blood pressure lowering is indicated.

8.3.1 Individual with uncomplicated hypertension

Recommendations

**The goal of therapy for simple hypertensive patients is blood pressure below 140/ 90 mm Hg.**	**A**

Supporting evidence

• The Hypertension Optimal Treatment (HOT) trial showed that the optimal target blood pressure in patients with a diastolic blood pressure of 100-115 mmHg was 139/83 mmHg[25].	1++

8.3.2 Patient with hypertension and diabetes

Recommendations

**Target blood pressure is below 130/80 mmHg.**	**A**

Supporting evidence

• Patients with hypertension and type 2 diabetes in the tighter blood pressure control group had far fewer cardiovascular system (CVS) events than those at the less tight control group[25].	1++
• The subgroup of diabetes patients randomised to lowest diastolic blood pressure group (<80 mmHg) had the most significant reductions in CVS events[25]. An even lower diastolic BP was found to be beneficial in diabetes patients	1++

8.3.3 Patient with hypertension and chronic kidney diseases

Recommendations

**Target blood pressure is below 130/80 mmHg.**	**A**

Supporting evidence

• Patients with chronic kidney disease in the low target blood pressure group had a significantly slower reduction in glomerular filtration rate (GFR) decline compared with patients assigned to the high target blood pressure group[26].	1++
• The risks for kidney failure and the composite outcome of kidney failure and all-cause mortality were significantly lower in the low target blood pressure group[27].	1++
• Systolic blood pressure range of 110-129 mmHg was associated with the lowest risk of kidney disease progression in patients with urine protein excretion > 1g/day[28].	1++

8.4 Follow up and monitoring

After initiating antihypertensive drug treatment, most patients should be followed up within 2 weeks until the blood pressure goal is achieved. More frequent visits may be indicated for patients with systolic blood pressure ≥ 160mmHg or diastolic blood pressure ≥ 100mgHg or with complications.

Once the blood pressure goal is achieved, the follow-up interval may be extended to 6-12 weeks depending on the patient’s condition and the doctor’s assessment.

To help monitor patient progress, it is recommended to have a clinical data base to capture key criteria such as:

- blood pressure,
- risk profile status and trend
- drug regimen,
- compliance,
- side effect,
- end organ assessment result, and
- specialist care.

In addition to routine follow-up, annual assessment is recommended for all patients under antihypertensive treatment. Module 8 provides details regarding annual assessment for people with hypertension.

8.5 Referral

Most patients with uncomplicated hypertension can be managed by their primary care practitioners. However, certain situations may warrant referral to specialists for assessment (Box 2).

Box 2. Referral to Specialists

Immediate referral to hospital setting

- Malignant hypertension
  - » DBP > 130mmHg
  - » Heavy proteinuria
  - » Papilloedema
  - » Encephalopathy
- Accelerated hypertension: DBP > 130mmHg and retinal hemorrhage
- Persistent BP > 220/120mmHg despite rest or drug treatment
- Pregnancy:
  - » BP ≥ 140/90mmHg and > 20 weeks gestation
  - » Signs and symptoms of pre-eclampsia (headache, proteinuria, oedema)

Referral to specialist
● Suspected secondary hypertension<br>● Patients aged 30 or below<br>● Hypertension in pregnancy of less than 20 weeks gestation without signs and symptoms of pre-eclampsia<br>● Patients with progressive complications e.g. target organ damage<br>● Medication problems for example:<br>» severe drug reaction<br>» treatment resistance<br>» multiple drug intolerance<br>» multiple drug contraindication<br>● Pregnancy

Chapter 9. Component 4: Patient Empowerment

Empowerment of patient requires an increase of their awareness about what they can do to prevent diseases occurrences in the first instance such as living healthier lifestyles, the need for regular health checks and also the need for self-maintenance, thereby sharing with their doctors the management of their chronic diseases such as diabetes and hypertension. Certainly, the healthcare professional needs to develop a working alliance with their patients to enhance and support their capacity for self-maintenance and self-care.

Patients should understand the nature of hypertension, the need for long-term medication and healthy lifestyle maintenance, the consequence of poor blood pressure control, and treatment options. They should also be informed of the possible side effects of drugs and to seek medical advice should they occur. The aim of patient education is to empower patients with the necessary knowledge and skills so that they can take charge of their own health, to adopt a healthy lifestyle and have better adherence to the management protocols (Box 3).

Written information should be given to patients if available e.g. pamphlets on healthy eating, techniques for taking blood pressure.

Box 3. Patient's Knowledge, Skill and Behaviour Checklist

**Patient with hypertension should know and practise:**
• Know the nature of the disorder<br>• Understand hypertension can be asymptomatic<br>• Know the risk of complications and, in particular, the importance of blood pressure monitoring<br>• Set individual target of treatment<br>• Know individual lifestyle requirements<br>• Know importance of exercise in treatment and practise regularly<br>• Know self-monitoring of blood pressure, and the meaning of blood pressure readings, as well as what action needs to be taken<br>• Know the possible side effects of drugs they are taking and seek doctor's advice promptly should they occur<br>• Need to regularly follow up with your doctors

Chapter 10. Future Direction To Promote The Use Of Reference Framework

The promulgation of the Reference Framework is a long-term and continuous process that entails multi-partite collaboration and multi-pronged strategies. To promote the adoption and implementation of the Reference Framework, a number of initiatives and pilot projects are planned or under way:

- A Clinical Advisory Group under the Task Force on Conceptual Model and Preventive Protocols of the Working Group on Primary Care has been established with members including experts from the academia, professional organizations, private and public primary care sectors and patient groups. The Clinical Advisory Group will review and update the Reference Framework regularly based on latest medical development and research.
- More details concerning the management of hypertension in primary care setting will be provided in the form of modules and will be available in the Primary Care Office website in 2011. The content will be updated as new evidence emerges.
- To facilitate the adoption of the Reference Framework, we are working with local healthcare providers of the private sector, professional organisations, non-government organisations and social service agencies in the community to develop and implement pilot projects to identify service gaps and provide affordable service as appropriate. In addition, primary care-related continuing medical education (CME) programmes for doctors will be organised with professional organisations.
- New service delivery models of care/ the concept of 'Community Health Centre' will be explored to foster the provision of more comprehensive and multidisciplinary primary care services, taking into consideration that more allied health and nursing support services are needed for management of patients with chronic disease like diabetes and hypertension.
- The patient education and empowerment is crucial for the adoption of the Reference Framework. We will work with relevant stakeholders to organise health promotional activities for this specific group of patients so that they can better understand the Framework and how it can help their condition.

Appendix 1. Smoking Cessation Services

Service	Organisation	Telephone number
Integrated Smoking Cessation Hotline of the Department of Health	Department of Health	1833 183 (Press 1)
Smoking Counselling and Cessation Hotline	Hospital Authority	1833 183 (Press 3), 2300 7272
Women Quit	The University of Hong Kong	2819 2692
Tung Wah Smoking Cessation Hotline	Tung Wah Group of Hospitals	1833 183 (Press 2), 2332 8977
Pok Oi Smoking Cessation Service using Traditional Chinese Medicine	Pok Oi Hospital	1833 183 (Press 4), 2607 1222

Acknowledgments

The Working Group on Primary Care gratefully acknowledges the invaluable contribution of the Members of the Task Force on Conceptual Model and Preventive Protocols and the Clinical Advisory Group on the Hong Kong Reference Framework for Hypertension Care in Adults in Primary Care Setting in the development of the Reference Framework.

Members of the Working Group on Primary Care of the Health and Medical Development Advisory Committee

**Chairman:**	
Dr York CHOW Yat-ngok	Secretary for Food and Health
**Alternate Chairman:**	
Prof Gabriel M LEUNG	Under Secretary for Food and Health
**Food and Health Bureau:**	
Ms Sandra LEE Suk-yee	Permanent Secretary for Food and Health (Health)
**Members:**	
Ms Elaine CHAN Sau-ho	Vice President, Group & Credit Insurance, Health Services Department, American International Assurance Company (Bermuda) Limited
Dr CHAN Wai-man	Assistant Director of Health (Family & Elderly Health Services), Department of Health
Dr Joseph CHAN Woon-tong	Deputy Medical Superintendent & Head, Department of Women's Health and Obstetrics, Hong Kong Sanatorium & Hospital
Dr Lincoln CHEE Wang-jin	Chief Executive Office , Quality Health Care Asia Limited
Dr Raymond CHEN Chung-i	Chief Executive Office , Hong Kong Baptist Hospital
Mr CHEUNG Tak-hai	Vice-chairperson, Alliance for Patients' Mutual Help Organizations

Dr CHU Leung-wing	Consultant & Chief Division of Geriatric Medicine, Queen Mary Hospital and Grantham Hospital<br>Hospital Authority
Dr Daniel CHU Wai-sing	Chief of Service and Cluster Service Coordinator (Family Medicine and Primary Healthcare) and Deputy Cluster Service Director (Community Services), Hong Kong East Cluster, Hospital Authority
Ms Ivis CHUNG Wai-yee	Chief Manager (Allied Health), Hospital Authority
Ms Sylvia FUNG Yuk-kuen	Chief Manager (Nursing) /Chief Nurse Executive, Hospital Authority
Prof Sian GRIFFITHS	Professor of Public Health & Director, School of Public Health and Primary Care, Faculty of Medicine, The Chinese University of Hong Kong
Ms Agnes HO Kam-har	Head of Medical and Group Life, HSBC Insurance (Asia) Limited
Dr Ronnie HUI Ka-wah	Chief Finance Officer and Executive Directo , Town Health International Holdings Co., Ltd.
Prof Cindy LAM Lo-kuen	Professor and Head, Department of Family Medicine and Primary Care, The University of Hong Kong
Ms Connie LAU Yin-hing	Chief Executive, Consumer Council
Dr Paco LEE Wang-yat	Specialist in Family Medicine<br>St. Paul's Hospital
Dr Sigmund LEUNG Sai-man	President, Hong Kong Dental Association
Dr Donald LI Kwok-tung	Specialist in Family Medicine<br>Director, Bauhinia Foundation Research Centre

Prof LIU Liang	Dean, School of Chinese Medicine, Hong Kong Baptist University
Dr LO Su-vui	Director (Strategy and Planning), Hospital Authority
Dr Louis SHIH Tai-cho	Specialist in Dermatology & Venereology
Dr TSE Hung-hing	Immediate Past President, The Hong Kong Medical Association
Dr Gene TSOI Wai-wang	Immediate Past President, The Hong Kong College of Family Physicians
Dr Nelson WONG Chi-kit	Head, Corporate Medical Scheme Service, Dr Vio & Partners
Prof Thomas WONG Kwok-shing	Vice President (Management), The Hong Kong Polytechnic University
Prof George WOO	Dean, Faculty of Health and Social Sciences, The Hong Kong Polytechnic University
Dr YEUNG Chiu-fat	President, Hong Kong Doctors Union

Members of the Task Force on Conceptual Model and Preventive Protocols

**Convener:**	
Prof Sian GRIFFITHS	Professor of Public Health & Director, School of Public Health and Primary Care, The Chinese University of Hong Kong
**Members:**	
Dr Alfred AU Si-yan	Service Director (Community Care), New Territories West Cluster, Hospital Authority
Prof Cecilia CHAN Lai-wan	Si Yuan Professor in Health and Social Work; Director, Centre on Behavioral Health; Professor, Department of Social Work and Social Administration, The University of Hong Kong

Dr CHAN Wai-man	Assistant Director of Health (Family & Elderly Health Services), Department of Health
Dr Joseph CHAN Woon-tong	Deputy Medical Superintendent & Head, Department of Women's Health and Obstetrics, Hong Kong Sanatorium & Hospital
Dr Lincoln CHEE Wang-jin	Chief Executive Office , Quality Health Care Asia Limited
Mr CHEUNG Tak-hai	Immediate Past Chairman & Vice-chairperson, Alliance for Patients' Mutual Help Organizations
Dr Regina CHING Cheuk-tuen	Assistant Director of Health (Health Promotion), Department of Health
Dr CHU Leung-wing	Consultant in-charge, Hong Kong West Cluster Geriatrics Service, Queen Mary Hospital, Hospital Authority
Dr Daniel CHU Wai-sing	Cluster Services Coordinator (Family Medicine & Primary Healthcare) & Consultant, Hong Kong East Cluster, Hospital Authority
Ms Ivis CHUNG Wai-yee	Chief Manager (Allied Health), Hospital Authority
Ms Sylvia FUNG Yuk-kuen	Chief Manager (Nursing) /Chief Nurse Executive, Hospital Authority
Dr Ronnie HUI Ka-wah	Finance (Executive) Director, Town Health International Holdings Co., Ltd
Prof Cindy LAM Lo-kuen	Professor and Head, Department of Family Medicine and Primary Care, The University of Hong Kong
Dr Augustine LAM Tsan	Chief of Service, Family Medicine, Prince of Wales Hospital; Cluster Co-ordinator (Community Partnership), New Territories East Cluster; Cluster Co-ordinator (Family Medicine), New Territories East Cluster, Hospital Authority

Dr Sigmund LEUNG Sai-man	President, Hong Kong Dental Association
Dr Shirley LEUNG Sze-lee	Principal Medical & Health Officer (Family Health Service), Department of Health
Dr LEUNG Ting-hung	Consultant Community Medicine (Non-Communicable Disease) and Head, Surveillance & Epidemiology Branch, Centre for Health Protection, Department of Health
Dr Donald LI Kwok-tung	Director, Bauhinia Foundation Research Centre
Prof LIU Liang	Dean, School of Chinese Medicine, Hong Kong Baptist University
Dr LO Su-vui	Director (Strategy and Planning), Hospital Authority
Dr Louis SHIH Tai-cho	Specialist in Dermatology & Venereology
Dr TSE Hung-hing	Immediate Past President, Hong Kong Medical Association
Dr Gene TSOI Wai-wang	Immediate Past President, The Hong Kong College of Family Physicians
Prof Thomas WONG Kwok-shing	Vice President (Management), The Hong Kong Polytechnic University
Dr Marcus WONG Mong-sze	Associate Consultant, Family Medicine and Primary Healthcare, Hong Kong East Cluster, Hospital Authority
Prof George WOO	Dean, Faculty of Health and Social Sciences, The Hong Kong Polytechnic University
Dr YEUNG Chiu-fat	President, Hong Kong Doctors Union

Dr Betty YOUNG Wan-yin	Cluster Chief of Service (Paediatrics & Adolescent Medicine), Hong Kong East Cluster, Hospital Authority; Chief of Service (Paediatrics & Adolescent), Pamela Youde Nethersole Eastern Hospital

Members of the Clinical Advisory Group on Reference Framework for Hypertension Care in Adults in Primary Care Setting

**Convener:**	
Prof Sian GRIFFITHS	Professor of Public Health & Director, School of Public Health and Primary Care, The Chinese University of Hong Kong
**Members:**	
Dr Amy CHAN Kit-ling	Private General Practitioner
Mr CHEUNG Tak-hai	Vice-Chairman, Alliance of Patient Mutual Help Organizations
Mr POON Kwok-fan	Chairman, The Hong Kong Stroke Association
Ms Caroline CHIU Wai-hing	Ward manager of New Territories East Cluster Family Medicine & General Outpatient Clinic, Fanling Family Medicine Centre
Dr Gabriel CHOI Kin	President, Hong Kong Medical Association
Dr Daniel CHU Wai-sing	Cluster Services Coordinator (Family Medicine & Primary Healthcare) & Consultant, Hong Kong East Cluster, Hospital Authority
Dr Ronnie HUI Ka-wah	Chief Finance Officer and Executive Directo , Town Health International Holdings Company Limited
Dr Linda HUI Yin-fun	Consultant (Elderly and Family Health Service), Department of Health

Prof Cindy LAM Lo-kuen	Professor and Head, Department of Family Medicine and Primary Care, The University of Hong Kong
Dr LAU Chu-pak	Specialist in Cardiology
Dr LAU Suet-ting	Co-Chairperson, Central Committee on Cardiac Service, Hospital Authority; Consultant, Medicine and Geriatrics, Princess Margaret Hospital
Prof Philip LI Kam-tao	Head of Division, Division of Nephrology, Department of Medicine and Therapeutics, The Chinese University of Hong Kong
Dr LI Sum-wo	Immediate Past President, The Association of Licentiates of Medical Council of Hong Kong
Dr Luke TSANG Chiu-yee	Consultant (Family Medicine), Department of Health
Prof TSE Hung-fat	Professor in Cardiology, Department of Medicine, The University of Hong Kong
Dr Bernard WONG Bun-lap	Specialist in Cardiology
Prof Martin WONG Chi-sang	Associate Professor, School of Public Health and Primary Care, The Chinese University of Hong Kong
Dr Chris WONG Kwok-yiu	President-Elect, Hong Kong College of Cardiology
Dr Michelle WONG Man-ying	Associate Consultant in Family Medicine and Primary Health Care, HK East Cluster, Hospital Authority
Dr Alexander WONG Shou-pang	Specialist in Cardiology
Dr YEUNG Chiu-fat	President, Hong Kong Doctors Union
Professor YU Cheuk-man	Head, Division of Cardiology, Department of Medicine and Therapeutics, The Chinese University of Hong Kong

Reference

1. U.S. Department of Health and Human Services. The seventh report of the Joint National Committee on Prevention, Detection, Evaluation, and Treatment of High Blood Pressure. [Internet]. Bethesda, MD: U.S. Department of Health and Human Services; c2004. Available from: http://www.nhlbi.nih.gov/guidelines/hypertension/jnc7full.pdf
2. Department of Health. Report on Population Health Survey 2003/04. Hong Kong SAR: Department of Health; 2005.
3. Nolte E, McKee M. Caring for people with chronic conditions – A health system perspective. European Observatory on Health Systems and Policies Series. Berkshire: Open University Press 2008.
4. Writing Group of the PREMIER Collaborative Research Group. Effects of comprehensive lifestyle modifications on blood pressure control: main results of the PREMIER clinical trial. JAMA. 2003; 289: 2083-2093.
5. The Royal Australian College of General Practitioners. Guidelines for preventive activities in general practice (the red book) 8th edition. [Internet]. South Melbourne (Australia): The Royal Australian College Of General Practitioners; c2012. Available from: http://www.racgp.org.au/your-practice/guidelines/redbook/
6. Kaplan N, Victor R. Kaplan's clinical hypertension. 10th ed: Philadelphia: Wolters Kluwer Health/Lippincott Williams & Wilkins; 2010.
7. National Institute for Health and Clinical Excellence (NICE). Hypertension: Clinical management of primary hypertension in adults. London (UK): NICE 2011. Available from: http://www.nice.org.uk/nicemedia/live/13561/56008/56008.pdf
8. Whelton PK, He J, Appel LJ, Cutler JA, Havas S, Kotchen TA, et al. Primary prevention of hypertension: clinical and public health advisory from The National High Blood Pressure Education Program. JAMA. 2002; 288:1882-8.
9. The Trials of Hypertension Prevention Collaborative Research Group. Effects of weight loss and sodium reduction intervention on blood pressure and hypertension incidence in overweight people with high-normal blood

pressure. The Trials of Hypertension Prevention, phase II. Arch Intern Med. 1997;157:657-67.

10. He J, Whelton PK, Appel LJ, Charleston J, Klag MJ. Long-term effects of weight loss and dietary sodium reduction on incidence of hypertension. Hypertension. 2000; 35:544-9.
11. Sacks FM, Svetkey LP, Vollmer WM, Appel LJ, Bray GA, Harsha D, et al. Effects on blood pressure of reduced dietary sodium and the Dietary Approaches to Stop Hypertension (DASH) diet. DASH-Sodium Collaborative Research Group. N Engl J Med. 2001;344 :3-10.
12. Vollmer WM, Sacks FM, Ard J, Appel LJ, Bray GA, Simons-Morton DG, et al. Effects of diet and sodium intake on blood pressure: subgroup analysis of the DASH-sodium trial. Ann Intern Med. 2001;135:1019-28.
13. Chobanian AV, Hill M. National Heart, Lung, and Blood Institute Workshop on Sodium and Blood Pressure: A critical review of current scientific evidence. Hypertension. 2000; 35:858-63.
14. Kelley GA, Kelley KS. Progressive resistance exercise and resting blood pressure: a meta-analysis of randomized controlled trials. Hypertension. 2000; 35:838-43.
15. Xin X, He J, Frontini MG, Ogden LG, Motsamai OI, Whelton PK. Effects of alcohol reduction on blood pressure: A meta-analysis of randomized controlled trials. Hypertension 2001;38:1112-7.
16. Fuchs FD, Chambless LE, Whelton PK, Nieto J, Heiss G. Alcohol consumption and the incidence of hypertension: the Atherosclerosis Risk in Communities Study. Hypertension 2001;37:1242-1250.
17. Thadhani R, Camargo CA, Jr, Stampfer MJ, Curhan GC, Willett WC, Rimm EB. Prospective study of moderate alcohol consumption and risk of hypertension in young women. Arch Intern Med 2002;162:569-574.
18. Anguilera MT, de la Sierra A, Coca A, Estruch R, Fernandez-Sola J, Urbano-Marquez A. Effect of alcohol abstinence on blood pressure: assessment by 24-hour ambulatory blood pressure monitoring. Hypertension 1999;33:653-657.

19. Doll R, Peto R, Boreham J, Sutherland I. Mortality in relation to smoking: 50 years' observations on male British doctors. BMJ 2004;328:1519-1533.
20. Kawachi I, Colditz GA, Stampfer MJ, Willett WC, Manson JE, Rosner B, et al. Smoking cessation and time course of decreased risks of coronary heart disease in middle-aged women. Arch Intern Med 1994;154:169-175.
21. Oncken CA, White WB, Cooney JL, Van Kirk JR, Ahluwalia JS, Giacco S. Impact of smoking cessation on ambulatory blood pressure and heart rate in postmenopausal women. Am J Hypertens 2001;14:942-949.
22. Bolinder G, de Faire U. Ambulatory 24-h blood pressure monitoring in healthy, middle-aged smokeless tobacco users, smokers, and nontobacco smokers. Am J Hypertens 1998;11:1153-1163.
23. Asia Pacific Cohort Studies Collaboration. Smoking, quitting, and the risk of cardiovascular disease among women and men in the Asia-Pacific region. Int J Epidemiol 2005;34:1036-45.
24. Hansson L. Zanchetti A. Carruthers SG. Dahlöf B. Elmfeldt D. Julius S, et al. Effects of intensive blood-pressure lowering and low-dose aspirin in patients with hypertension: principal results of the Hypertension Optimal Treatment (HOT) randomised trial. HOT Study Group. Lancet 1998;351:1755-62.
25. Tight blood pressure control and risk of macrovascular and microvascular complications in type 2 diabetes: UKPDSA 38. UK Prospective Diabetes Study Group. BMJ 1998;317:703-13.
26. Peterson JC, Adler S, Burkart JM, Greene T, Hebert LA, Hunsicker LG, et al. Blood pressure control, proteinuria, and the progression of renal disease. The Modification of Diet in Renal Disease Study. Ann Intern Med 1995;123:754.
27. Sarnak MJ, Greene T, Wang X, Beck G, Kusek JW, Collins AJ, et al. The effect of a lower target blood pressure on the progression of kidney disease: Long-term follow-up of the modification of diet in renal disease study. Ann Intern Med 2005;142:342-51.
28. Jafar TH, Stark PC, Schmid CH, Landa M, Mascho G, de Jong PE, et al. Progression of chronic kidney disease: The role of blood pressure control, proteinuria, and angiotensin-converting enzyme inhibition: a patient-level meta-analysis. Ann Intern Med 2003;139:244-52.

Module 1 Framework for Population Approach in the Prevention and Control of Hypertension across the Life Course

Age group	Lifestyle advice	Risk assessment	Screening	Disease management	Complication monitoring	Rehabilitation care
**Antenatal**	• A balanced diet<br>• Regular intake of carbohydrates<br>• Lower in fat<br>• Plenty of fruits and vegetables<br>• Physically active	• Monitor weight gain	• Watch out for pre-eclampsia	• Early antenatal care<br>• Blood pressure and lipid control<br>• Self-care	• Monitor fetal growth<br>• Obstetric complications in women	
**Infancy**	• Breast feeding<br>• Avoid obesity<br>• Regular exercise<br>• Adequate sleep	• Monitor weight gain				
**Childhood**	• Abstain from smoking<br>• Regular exercise<br>• Healthy eating habit<br>• Limit sodium intake	• Monitor BMI	• Watch out for secondary hypertension	• Treat secondary hypertension<br>• Monitor growth and development<br>• Carer education and support	• Growth and development<br>• Malignant hypertensions<br>• Hypotension	
**Adulthood**	• Abstain from smoking<br>• Smoking cessation for smokers<br>• Healthy eating habit<br>• Limit sodium intake<br>• Weight management<br>• Regular exercise<br>• Limit alcohol consumption	• Monitor BMI<br>• Monitor abdominal circumference<br>• Family history of diabetes	• Measure blood pressure for all individuals aged ≥ 18 every 2 years[1]<br>• More frequent blood pressure measurement for individuals with moderate or high risk of vascular disease[1]<br>• Opportunistic measurement of blood pressure at all clinic visits	• Blood pressure and lipid control<br>• Monitor the adverse effect of drug treatment<br>• Self-care	• Target organ damage, e.g. nephropathy, neuropathy, cardiovascular complicationss<br>• Hypotension<br>• Malignant hypertension	

Age group	Lifestyle advice	Risk assessment	Screening	Disease management	Complication monitoring	Rehabilitation care
**Elderly**	• Abstain from smoking<br>• Smoking cessation for smokers<br>• Healthy eating habit<br>• Limit sodium intake<br>• Weight management<br>• Regular exercise	• Monitor BMI<br>• Monitor abdominal circumference<br>• Diabetes	• Measure blood pressure as above-mentioned	• Blood pressure and lipid control<br>• Beware of increased risk of hypotension in elderly<br>• Monitor the adverse effect of drug treatment<br>• Self-care<br>• Carer education and support	• Hypotension<br>• Malignant hypertension<br>• Target organ damage: nephropathy, neuropathy, cardiovascular complications	• Optimise patient's potential to cope with hypertension and its complications. Example: myocardial infarction, cerebrovascular disease, nephropathy, etc.<br>• Provide support to carer and loved ones<br>• Multidisciplinary approach in rehabilitation for stroke and renal failure

Reference:

1. The Royal Australian College of General Practitioners. Guidelines for preventive activities in general practice (the red book) 8th edition. [Internet]. South Melbourne (Australia): The Royal Australian College Of General Practitioners; c2012. Available from: http://www.racgp.org.au/your-practice/guidelines/redbook/

1. Equipment for recording blood pressure

1.1 Sphygmomanometer

i. Mercury sphygmomanometer – the most reliable type of instrument for recording blood pressure. However, mercury is being gradually phased out due to health and environmental hazard[1]. WHO recommends that mercury sphygmomanometers be retained for calibration purposes in designated laboratories[2].

ii. Electronic devices – can also be used, but periodic calibration should be done to ensure its accuracy.

iii. Electronic devices that record the pressure in the fingers or the wrist should be avoided.

1.2 Checking of mercury sphygmomanometer

i. The column of the manometer is in the intended position (vertical).

ii. Mercury level is at zero when cuff is deflated.

iii. No blockage of the air venting system at the top of the manometer

iv. A sluggish response or bouncing of the mercury column during inflation and deflation usually indicates a blocked vent.

v. No leakage from rubber tubing, hand pump and control valve:

a. Roll a cloth cuff into its own tail.

b. Pump up to 200 mmHg and wait for 10 seconds.

c. Mercury should fall < 2 mmHg in 10 seconds.

d. If fall > 2 mmHg, clamp circuit in sections to locate the leakage or replace the control valve.

1.3 Checking of electronic devices

i. Routine checks - compare the reading with mercury sphygmomanometer.

ii. Periodic calibration is needed.

iii. If consistent discrepancies of more than 5 mmHg persist, refer to service manual or send the monitor to a trained technician for calibration.

2. Blood pressure recording techniques

i. The client should be advised to be seated for at least 5 minutes before the recording is taken.

ii. Arrange client in sitting position.

iii. Remove any constrictive clothing from the arm.

iv. Support client's arm with the antecubital fossa at heart level.

v. Use an appropriate sized blood pressure cuff. The cuff should be wide enough to cover two thirds of the upper arm and its length should be long enough to encircle the whole arm.

vi. Advise client to relax and not to talk during blood pressure recording.

vii. Check blood pressure initially by palpation prior to auscultation.

   a. palpate the radial artery with your fingertips.
   b. inflate the cuff while simultaneously palpating the artery.
   c. note the point on the manometer at which the radial artery pulsation is no longer palpable. (This is the estimated systolic pressure.)
   d. deflate the cuff.

viii. Wait 30-60 seconds before reinflating.

ix. Place the stethoscope gently over the brachial artery and steadily inflate the cuff to the level of 30 mmHg above the estimated level of systolic pressure checked by palpation.

x. Deflate the blood pressure cuff by 2 mmHg per second.

xi. Record the first Korotkoff sound (the regular appearance of sound) as the systolic pressure.

xii. Record the last (5th) Korotkoff sound (the disappearance of sound) as the diastolic pressure. If sounds persist to zero, or close to zero, use the muffling sounds (4th Korotkoff sound) to indicate diastolic pressure.

xiii. Allow 1 minute between blood pressure recordings.

xiv. Before using an electronic device, check for pulse irregularity. If pulse irregularity is present, measure blood pressure manually using direct auscultation over the brachial artery[3].

3. Precautions about blood pressure recording

3.1 Recorder's precautions

i. Read at eye level.

ii. Avoid digital preference. The blood pressure reading should be corrected to the nearest 2 mmHg.

iii. Choose the correct cuff size (see 2 v).

iv. Consistent use of the 4th or 5th Korotkoff sounds for recording (see 2 xii).

v. Correct arm positioning

  a. blood pressure changes 8-10 mmHg for every 10 cm that the antecubital fossa is above or below the heart level.

  b. arm well supported (diastolic pressure may be raised by as much as 10%).

vi. Deflate the cuff not too rapidly or too slowly (see 2 x).

vii. Avoid venous congestion due to repeated measurement.

viii. Adopt a unified standard in recording routinely to avoid variation among recorders.

3.2 Patient's factors

i. Emotional factors including white coat hypertension: 24-hour ambulatory blood pressure monitoring and self BP monitoring at home can be used to address the white coat effect.

ii. Physical exertion: blood pressure will increase during exertion.

iii. After exercise, decrease in blood pressure may persist for more than one hour.

iv. After meals: blood pressure may decrease following meals; recording is not recommended within half an hour of eating.

v. Smoking and caffeine: should be avoided within 30 minutes prior to BP recording[4,5].

vi. Alcohol.

vii. Temperature extremes.

viii. Bladder and bowel distension.

ix. Pain.

4. Home/ self BP monitoring

4.1 Potential advantages and disadvantages of home monitoring

Advantages	***Disadvantages***
➢ Providing information on response to antihypertensive medication<br>➢ Improving patient adherence with therapy[6]<br>➢ Evaluating	➢ Reporting bias<br>➢ Unsupervised alteration of medication

4.2 Self-measurement of BP

- ➢ Persons with an average BP 135/85 mmHg measured at home are generally considered to be hypertensive[5].
- ➢ Two consecutive measurements are taken, at least 1 minute apart and with the person seated and blood pressure is recorded twice daily[3].
- ➢ Initial assessment or the assessment of treatment effects should be for a 7-day period, with recordings performed in the morning and evening, and excluding values for the first 24 hours. The average of the readings is taken as the home BP level.
- ➢ Home measurement devices should be checked regularly.

Reference:

1. Minamata Convention Agreed by Nations. [Internet] [updated 19 Jan 2013; cited 18 Jun 2013]. Available from: http://unep.org/newscentre/Default.aspx?DocumentID=2702&ArticleID=9373&l=en

2. World Health Organization. Information provided by WHO on mercury in health care, related WHO activities, resources and risk assessment methodologies. [Internet]. Available from: http://www.unep.org/hazardoussubstances/Portals/9/Mercury/Documents/INC1/WHO%20Information%20on%20Mercury.pdf

3. National Institute for Health and Clinical Excellence. Hypertension: Clinical management of primary hypertension in adults. London(UK): National Institute for Health and Clinical Excellence; c 2011 [cited 13 Feb 2012]. Available from: http://www.nice.org.uk/nicemedia/live/13561/56008/56008.pdf

4. American Heart Association. Instructional Video - Monitoring Blood Pressure at Home. [Internet]. Texas (United States): American Heart Association; 2012. Available from: http://www.heart.org/HEARTORG/Conditions/HighBloodPressure/SymptomsDiagnosisMonitoringofHighBloodPressure/Instructional-Video---Monitoring-Blood-Pressure-at-Home_UCM_303324_Article.jsp

5. U.S. Department of Health and Human Services. The seventh report of the Joint National Committee on Prevention, Detection, Evaluation, and Treatment of High Blood Pressure. [Internet]. Bethesda, MD: U.S. Department of Health and Human Services; 2004. Available from: http://www.nhlbi.nih.gov/guidelines/hypertension/jnc7full.pdf

6. American Heart Association. Home blood pressure monitoring [Internet]. Texas (United States): American Heart Association, c2011 [updated 21 Jan 2011; cited 24 May 2011]. Available from: http://www.heart.org/HEARTORG/Conditions/HighBloodPressure/SymptomsDiagnosisMonitoringofHighBloodPressure/Home-Blood-Pressure-Monitoring_UCM_301874_Article.jsp

Table 1. Identifiable causes of hypertension (the ABCDE mnemonic)

**A**	• **A**ccuracy of diagnosis<br>• obstructive sleep **A**pnea
**B**	• renal artery **B**ruits (renovascular disease noise)<br>• **B**ad kidneys (renal parenchymal disease)
**C**	• excess **C**atecholamines<br>• **C**oarctation of the aorta<br>• **C**ushing's syndrome
**D**	• **D**rugs (immunosuppressive agents, NSAID, COX-2 inhibitors, estrogens / oral contraceptive, weight-loss agents, stimulants, mineralocorticoids, antiparkinsonian, monoamine oxidase inhibitors, anabolic steroids, sympathomimetics)<br>• **D**iet (high salt intake, excessive alcohol intake, obesity)
**E**	• excess **E**rythropoietin<br>• **E**ndocrine disorders (phaeochromocytoma, primary and secondary hyperaldosteronism, hyper- or hypothyroidism, parathyroid disease, Cushing syndrome)

Table 2. Findings suggestive of secondary hypertension

**Findings**	**Disorder suspected**
Snoring, daytime somnolence, obesity	Obstructive sleep apnea
Hypernatremia, hypokalemia, increased urinary excretion of potassium	Hyperaldosteronism
Renal artery bruits, renal insufficiency, atherosclerotic cardiovascular disease, oedema, elevated blood urea nitrogen and creatinine levels, proteinuria	Renal parenchymal disease
Systolic/diastolic abdominal bruit	Renovascular disease
Use of sympathomimetics, perioperative setting, acute stress, tachycardia	Excess catecholamines
Decreased or delayed femoral pulses, abnormal chest radiograph	Coarctation of aorta

Findings	Disorder suspected
Weight gain, fatigue, weakness, hirsutism, amenorrhea, moon facies, dorsal hump, purple striae, truncal obesity, hypokalemia	Cushing's syndrome
Use of drug (immunosuppressive agents, NSAID, COX-2 inhibitors, estrogens / oral contraceptive, weight-loss agents, stimulants, mineralocorticoids, antiparkinsonian, monoamine oxidase inhibitors, anabolic steroids, sympathomimetics)	Side effects of drugs
High salt intake, excessive alcohol intake, obesity	Unhealthy diet
Erythropoietin use in renal disease, polycythemia in chronic obstructive pulmonary disease (COPD)	Erythropoietin side effect
Paroxysmal hypertension, headaches, diaphoresis, palpitations, tachycardia	Phaeochromocytoma
Fatigue, weight gain, hair loss, diastolic hypertension, muscle weakness	Hypothyroidism
Heat intolerance, weight loss, palpitation, systolic hypertension, exophthalmos, tremor, tachycardia	Hyperthyroidism
Kidney stones, osteoporosis, depression, lethargy, muscle weakness	Hyperparathyroidism
Headaches, fatigue, visual problems, enlargement of hands, feet, tongue	Acromegaly

Reference:

1. Hebert PR, Moser M, Mayer J, Glynn RJ, Hennekens CH: Recent evidence on drug therapy of mild to moderate hypertension and decreased risk of coronary heart disease. Arch Intern Med. 1993;153(5):578-81.

2. Onusko E. Diagnosing Secondary Hypertension. Am Fam Physician. 2003; 67(1):67-74.

Module 4 Evaluation for All Newly Diagnosed Hypertensive Patients[1]

The aims of the evaluation are:

- to assess lifestyle and identify cardiovascular risk factors that may affect prognosis and guide treatment,
- to reveal identifiable causes of high blood pressure, and
- to assess the presence or absence of target organ damage and cardiovascular disease. (Box 1)

Box 1 Target organ damage[2]

- Heart: Left ventricular hypertrophy (ECG / Echocardiogram)
- Renal: Albuminuria and / or elevation of plasma creatinine (>110 µmol/L)
- Vessel: USG or radiological evidence of atherosclerotic plaque (carotid, iliac, femoral or aorta), generalised or focal narrowing of the retinal arteries
  - Angina or prior myocardial infarction
  - Prior coronary revascularisation
  - Heart failure
- Brain:
  - Stroke or transient ischaemic attack
  - Dementia
- Chronic renal disease
- Peripheral arterial disease
- Retinopathy: Hemorrhages or exudates, Papilloedema

History

- Symptoms suggestive of secondary causes
- Symptoms (present or past history) of cardiovascular risk factors: age>55 for men and age>65 for women/ smoking/ raised total cholesterol/ diabetes mellitus/ family history of premature cardiovascular disease (men under age 55 or women under age 65)
- Target organ damage: retinopathy/ left ventricular hypertrophy/ heart failure/ coronary heart disease (CHD)/ proteinuria or renal disease like renal failure / peripheral vascular disease (PVD)/ cerebrovascular accident (CVA)
- Factors that affect choice of drug treatment like gout, bronchospasm.
- Assessment of lifestyle: exercise, diet, alcohol
- Drug history
- Psychosocial factors
- Family history: hypertension (HT), diabetes, stroke, renal disease, myocardial infarction and other cardiovascular system (CVS) risk factors

Physical examination

- Body weight, height, Body Mass Index
- Features of Cushing’s syndrome / Acromegaly (endocrine case of HT)
- Skin stigmata of neurofibromatosis (phaeochromocytoma)
- Radial/brachial femoral delay, precordial or chest murmurs (aortic coarctation or aortic disease)
- Abdominal examination: enlarged kidney, abdominal bruit
- Signs of organ damage: peripheral arterial disease, fundi abnormalities, carotid bruit, neurological defects, CVS examination

Routine laboratory investigation

Recommended Tests	***Justification***
General	
Urine analysis	• Detection of hypertensive nephropathy<br>• Look for red blood cells, casts, glucose and protein
Fasting glucose level	• Detection of concomitant impaired glucose tolerance and diabetes
Renal function test	
Sodium, Potassium, Urea, Creatinine	• Baseline level of electrolytes to aid adjustment of future pharmacotherapy (e.g. thiazide, beta-blockers, Angiotensin-converting enzyme inhibitors<br>• Detection of hypertensive nephropathy<br>• Detection of electrolytes disturbance associated with endocrine disorders
Lipid profile	
Total Cholesterol<br>Triglyceride<br>HDL-Cholesterol<br>LDL-Cholesterol	• Detection of concomitant cardiovascular risk factors<br>• Guidance for antihypertensive and lipid-lowering pharmacotherapy
ECG	
12-lead electrocardiogram	• Detection of cardiac complication as possible end-organ damage (left ventricular hypertrophy; cardiac ischemia)

Reference:

1. U.S. Department of Health and Human Services. The seventh report of the Joint National Committee on Prevention, Detection, Evaluation, and Treatment of High Blood Pressure. [Internet]. Bethesda, MD: U.S. Department of Health and Human Services; c2004 [cited 24 May 2011]. Available from: http://www.nhlbi.nih.gov/guidelines/hypertension/jnc7full.pdf

2. The Royal Australian College of General Practitioners. Guidelines for preventive activities in general practice 7th edition. [Internet]. South Melbourne (Australia): The Royal Australian College of General Practitioners; c2009 [cited 24 May 2011]. Available from: http://www.racgp.org.au/redbook/index

The Dietary Advice to Stop Hypertension eating plan[1]

The Dietary Advice to Stop Hypertension (DASH) eating plan, developed by United States National Institute of Health, features plenty of fruits, vegetables, whole grains, and other foods that are heart healthy and lower in salt/ sodium. It is recommended for people with high blood pressure. Please refer to Table 1 for DASH eating plan.

The DASH eating plan has the following characteristics:

1. Rich in fruits, vegetables
2. Rich in potassium, magnesium and calcium
3. Low in cholesterol, saturated and total fat
4. Low in sodium
5. Low in sweets and added sugars

Table 1. The DASH eating plan shown below is based on 2000 calories a day. The number of daily servings in a food group one requires may vary from those listed, depending on the caloric needs.

**Food Group**	**Daily Servings**	**Examples of Serving Sizes**
Grain and grain products	6-8	1 slice bread<br>1 cup[Note] ready-to-eat cereal<br>1/2 cup cooked rice, pasta, or cereal
Vegetables	4-5	1 cup raw leafy vegetables<br>1/2 cup cooked vegetables<br>1/2 cup vegetable juice
Fruits	4-5	1 medium fruit<br>1/4 cup dried fruit<br>1/2 cup fresh, frozen, or canned fruit<br>1//2 cup fruit juice
Low fat or fat free dairy products	2-3	1 cup milk<br>1 cup yoghurt<br>1 1/2 ounce cheese
Lean meats, poultry, and fish	6 or less	1 ounce (28 grams) cooked lean meat, skinless poultry, or fish

Food Group	Daily Servings	Examples of Serving Sizes
Nuts, seeds, and dry beans	4-5 per week	1/3 cup or 1 1/2 ounces nuts<br>1 tablespoon or 1/2 ounces seeds
Fats and oils	2-3	1 teaspoon soft margarine<br>1 tablespoon low-fat mayonnaise<br>2 tablespoons light salad dressing<br>1 teaspoon vegetable oil
Sweets	5 or less per week	1 tablespoon sugar<br>1 tablespoon jelly or jam<br>1 cup lemonade

Note: Volume of cup = 240 ml

Specific instructions on dietary intervention (adapted from DASH eating plan)

Salt

Restrict salt intake to less than 5 grams of table salt per day (around 1 teaspoon), as sodium is a main dietary factor for elevated blood pressure. The 5 grams includes all salt and sodium consumed, including that used in cooking and at the table. Please refer to table 2 for practical advice.

Tips to reduce salt and sodium

- Compare nutrition labels and choose prepackaged foods that are lower in sodium.
- Use fresh poultry, fish, lean meat and vegetables. Limit consumption of foods that are high in salt content such as sauces, preserved meat and vegetables, and canned and processed foods like luncheon meat and sausages.
- Cut down on convenient and instant foods, canned soups or broths as they often contain a lot of sodium.
- Cook food with less salt. Use spices instead of salt in cooking and at the table. Limit condiments, such as mustard, ketchup, pickles and soy sauce.
- Use more natural low-sodium seasoning, such as ginger, green onions, garlic, pepper powder, lemon juice, etc. to replace salt or soy sauce in cooking.

Salt substitutes

- A potassium-rich diet may help to reduce blood pressure.
- Potassium should be from food sources, not from supplements.
- Many fruits and vegetables e.g. potato, spinach, tomato, lettuce, banana, orange, apple and some dairy products e.g. yoghurt, and fish are rich sources of potassium.
- However, potassium-rich diet should be avoided in patients with chronic renal failure or taking potassium-sparing diuretics.

Table 2. Practical advice for low salt diet

**Meals and foods**
**Breakfast**<br>• 1 cup oatmeal<br>• 2 slice whole wheat bread<br>• 2 teaspoons of soft margarine<br>• 1 cup low fat or skimmed milk<br>• 1 medium fruit
**Lunch**<br>• 2 cups cooked rice or pasta<br>• 3 ounces (84 grams) of cooked meat, fish or poultry<br>• 1/8 teaspoon of salt in cooking, or equivalent<br>• 1 teaspoon vegetable oil<br>• 1 cup cooked vegetable<br>• 1 medium fruit
**Dinner**<br>• 1 medium baked potato<br>• 1 teaspoon of sour cream<br>• 3 ounces (84 grams) of cooked meat, fish or poultry<br>• 1/8 teaspoon of salt in cooking, or equivalent<br>• 1 teaspoon vegetable oil<br>• 1 cup cooked vegetable<br>• 1 medium fruit

Meals and foods
**Snack**<br>• 1 medium fruit<br>• 1 slice whole wheat bread<br>• 1.5 ounces reduced fat cheddar cheese, low sodium<br>• 1 cup low fat yoghurt
**Total salt used per day: 1/4 teaspoon of salt**

Note : Salt exchange list: 1/8 teaspoon of salt = 1/2 teaspoon of soya sauce
= 1 oyster sauce
= 1 tablespoon of Ketchup

Carbohydrate intake[2]

- Carbohydrate should provide half of the total energy intake.
- Meals should contain mostly complex carbohydrates with an emphasis on high-fibre foods such as vegetables, whole grain cereals and fruits.
- Simple sugar including sugar sweetened beverages (e.g. soft drinks, fruit juice) and snacks with high sugar content (e.g. cakes) should provide no more than 10% of total energy intake.

Fruit and Vegetables

- Include 4-5 servings of fruit a day.
- Eat at least 4-5 servings of vegetables a day.
- Choose fruit more often than juice.
- Choose a wide variety of fruit and vegetables.

Protein[2]

- Protein should provide 15-20% of total energy intake.
- Good sources of protein are fish, seafood, lean meat, chicken, low fat dairy products, nuts and soy bean products which have not been deep-fried.

Fat[2]

- No more than 30% of total energy intake should come from fat.
- Cut down on foods with high saturated fat.
  - High-fat meat and meat products, such as bacon, spare ribs, sausages, and canned meat like luncheon meat.
  - High-fat poultry parts, such as chicken skin and chicken wings.
  - High-fat dairy products, such as ice-cream, whole milk and cheese made from whole milk.
  - Fat and oil, such as butter, cream, lard and chicken fat.
  - High-fat foods from plants: Palm oil, coconut, and coconut products, such as coconut oil and coconut milk.
- Cut Down on Foods High in Cholesterol. Foods high in cholesterol include:
  - Offal, such as brain, liver and kidney.
  - Seafood, such as octopus, squid, fish head, and roe of shrimp and crab.
  - Egg yolk is high in cholesterol but also rich in nutrients and can be part of a healthy diet. If a person has an egg for breakfast, he/she might want to substitute some vegetarian choices for their other meals of the day.
- Cut Down on Foods that Contain Trans Fat. Foods high in Trans Fat: Hard margarine, cookies, cakes, croissants, French fries, potato chips and doughnuts.
- Use vegetable oil such as canola oil and peanut oil for cooking instead of animal fat.

Alcohol Consumption

- Consumption should be limited to less than 2 standard drinks for men and less than 1 standard drink for women every day.
- Each standard drink contains 10 grams of pure alcohol. Defining one standard drink as 10 grams of pure alcohol, it equates to about 250 ml of regular beer at 5% of alcohol content, one small glass (100 ml) of wine at 12% alcohol content, or one pub measure (30ml) of hard liquor at 40% of alcohol content.

Reference:

1. National Heart, Lung, and Blood Institute. Your Guide to Lowering Your Blood Pressure With DASH. [Internet]. (US): National Heart, Lung, and Blood Institute; c2006[cited 24 May 2011]. Available from: http://www.nhlbi.nih.gov/health/public/heart/hbp/dash/new_dash.pdf

2. Department of Health. Heart Healthy Diet. [Internet]. (Hong Kong): Department of Health; c2006 [updated 1 October 2006; cited 24 May 2011]. Available from: http://www.info.gov.hk/elderly/english/healthinfo/lifestyles/heart_healthy_diet-e.htm

Module 6 Exercise Recommendations to People with Hypertension (Adopted from Department of Health Central Health Education Unit Exercise Prescription 2011 Edition)

1. Effect of Exercise

Epidemiological studies suggest that regular physical activity may be beneficial for both prevention and treatment of hypertension, to enable weight loss, for functional health status, and to diminish all-cause mortality and risk of cardiovascular disease. Cross-sectional studies of select populations from China and other Eastern populations have confirmed the presence of a strong association between physical inactivity and an adverse heart disease risk factor profile[1]. In Japanese men, duration of walk-to-work and leisure-time physical activity was significantly associated with a reduction in the risk for incident hypertension[2]. A meta-analysis of randomized controlled trials concluded that dynamic aerobic endurance training reduces resting systolic and diastolic blood pressures by 3.0/2.4 mmHg, and daytime ambulatory blood pressure by 3.3/3.5 mmHg. The reduction in resting blood pressure was more pronounced in the hypertensive group (-6.9/-4.9 mmHg) than in the normotensive group (-1.9/-1.6 mmHg)[3]. Even moderate levels of exercise lowered blood pressure, and this type of exercise also reduced body weight, body fat and waist circumference[4]. Dynamic resistance exercise can also decrease resting blood pressure by 3.5/3.2 mmHg[5].

2. Recommendations for Exercise Prescription

The following table summarises the exercise prescription that is recommended for patients with hypertension in general.

Physical Activity Profile	Recommendations*
Frequency	• Perform aerobic exercise preferably all days of the week[6].<br>• Supplemented by resistance exercise twice to thrice weekly on nonconsecutive days[6].

* Given that many patients may present with comorbidities, it may be necessary to tailor the exercise prescription accordingly.

Physical Activity Profile	Recommendations*
Intensity	• Aerobic exercise should be at least at moderate intensity (e.g. brisk walking), corresponding approximately to 40-60% of maximal aerobic capacity ($VO_{2\ max}$)[6]. Relatively, moderate-intensity activity could be expressed as a level of effort of 5 or 6 on a scale of 0 to 10 (where 0 is the level of effort of sitting, and 10 is maximal effort) or 50–70% of maximum heart rate[7,8].<br>• Resistance exercise should be at moderate intensity[6], which could be expressed as 50-70% of 1-repetition maximum (1-RM– maximum amount of weight one can lift in a single repetition for a given exercise) [8].
Time	• Perform 30 to 60 min per day of aerobic exercise continuously or intermittently in bouts of at least 10 min accumulated to total of at least 30 min per day[6].<br>• Each session of resistance exercise should minimally include 8–10 exercises and should consist of at least 1 set of 8–12 repetitions per exercise[6].
Type	• Emphasis on aerobic exercises such as walking, jogging, cycling and swimming[6]. Rope skipping is also a very good option that can be performed every day, requires little equipment and learning, and involves a lot of muscle group. However, any activity that uses large muscle groups, can be maintained continuously, and is rhythmical and aerobic in nature is recommended as the primary modality for those with hypertension [9].<br>• Resistance exercise should involve the major muscle groups (legs, hips, chest, back, abdomen, shoulders, and arms)[6]. Either machine weights or free weights might be used while the former is likely the safest approach[10]. Resistance exercise performed should be alternating between upper- body and lower-body works to allow for adequate rest between exercises. Some examples of resistance exercise include chest press, shoulder press, triceps extension, biceps curl, pull-down (upper back), lower-back extension, abdominal crunch/curl-up, quadriceps extension or leg press, leg curls (hamstrings), and calf raise[10].

Regular physical activity of even lower intensity and duration, however, has been shown to be associated with about a 20% decrease in mortality in cohort studies[11]. Individuals engaging in resistance exercise should seek guidance by a trained professional, for appropriate machine adjustment, selection of specific exercises, appropriate initial exercise prescription, and subsequent exercise progression[10]. Resistive isotonic activities, when done as the only form of exercise training, are not recommended for lowering blood pressure in hypertensive patients[12]. An exercise prescription for achieving and maintaining flexibility, such as proper stretching for all the major joints, may be advised after a thorough warm-up and during the cool-down period[6].

3. Rate of Progression

In November 2010, the American College of Sports Medicine and the American Diabetes Association published a joint position statement on exercise recommendations for patients with Type 2 diabetes mellitus which covers rate of progression[13]. Their general principles, as outlined below, can also be applied to patients with hypertension:

- To avoid injury, progression of frequency and intensity of resistance exercise should occur slowly[13].
- Gradual progression of intensity of aerobic exercise is also advisable to enhance compliance[13].

4. Evaluation of patient with hypertension before recommending an exercise programme

The need for and scope of pre-exercise evaluation of the cardiovascular status will depend on the extent of the envisaged exercise and on the patient's symptoms and signs, total cardiovascular risk and associated clinical conditions[14]. The risk of cardiovascular disease in patients with hypertension is determined not only by the level of blood pressure, but also by the presence or absence of target organ damage and other risk factors such as smoking, dyslipidaemia and diabetes, as shown in

the Seventh Report of the Joint National Committee on Prevention, Detection, Evaluation, and Treatment of High Blood Pressure[15]. These factors independently modify the risk for subsequent cardiovascular disease, and their presence or absence is determined during the routine evaluation of patients with hypertension (i.e., history, physical examination, and/or laboratory tests). High-intensity resistance training should not be initiated for persons without prior exposure to more moderate resistance exercise independently of age, health status, or fitness level[10]. Therefore, patients with hypertension should consult a primary care practitioner prior to any substantive increase in physical activity, particularly vigorous-intensity activity[16].

5. Special Precautions

1. Intensive isometric exercise such as heavy weight lifting can have a marked pressor effect and should be avoided[14].
2. If hypertension is poorly controlled, heavy physical exercise as well as maximal exercise testing should be discouraged or postponed until appropriate drug treatment has been instituted and blood pressure lowered[16]. When exercising, it appears prudent to maintain systolic blood pressures at ≤220 mmHg and/or diastolic blood pressures ≤105 mmHg[6].
3. β-blockers and diuretics may adversely affect thermoregulatory function and cause hypoglycaemia in some individuals. In these situations, educate patients about the sign and symptoms of heat intolerance and hypoglycaemia, and the precautions that should be taken to avoid these situations[6].
4. Antihypertensive medications such as calcium channel blockers, α-blockers and vasodilators may lead to sudden reductions in post-exercise blood pressure. Extend and monitor the cool-down period carefully in these situations[6].
5. β-blockers, particularly the non-selective types, may reduce sub-maximal and maximal exercise capacity primarily in patients without myocardial ischaemia. Consider using perceived exertion to monitor exercise intensity in these individuals[6].
6. Patients should be informed about the nature of cardiac prodromal symptoms e.g. shortness of breath, dizziness, chest discomfort or palpitation and seek prompt medical care if such symptoms develop.

Reference:

1. Hong Y, Bots ML, Pan X, Wang H, Jing H, Hofman A, et al. Physical activity and cardiovascular risk factors in rural Shanghai, China. Int J Epidemiol. 1994;23(6):1154-58.

2. Hayashi T, Tsumura K, Suematsu C, Okada K, Fujii S, Endo G. Walking to work and the risk for hypertension in men: the Osaka Health Survey. Ann Intern Med. 1999;131(1):21-6.

3. Cornelissen VA, Fagard RH. Effects of endurance training on blood pressure, blood pressure-regulating mechanisms, and cardiovascular risk factors. Hypertension. 2005;46(4):667-75.

4. Fagard RH. Exercise characteristics and the blood pressure response to dynamic physical training. Med Sci Sports Exerc 2001;33(6 Suppl):S484-92.

5. Cornelissen VA, Fagard RH. Effect of resistance training on resting blood pressure: a meta-analysis of randomized controlled trials. J Hypertens 2005;23(2):251-9.

6. Exercise Prescription for Other Clinical Populations. In: ACSM's guidelines for exercise testing and prescription. 8th edition. Walter R Thompson; Neil F Gordon; Linda S Pescatello; American College of Sports Medicine. Philadelphia(US): Wolters Kluwer Health/Lippincott Williams & Wilkins; 2010. p. 249

7. Physical Activity Guidelines Advisory Committee. Physical Activity Guidelines Advisory Committee Report, 2008. [Internet]Washington (US); US Department of Health and Human Services; c2008.[cited 25 May 2011]. Available from: http://www.health.gov/paguidelines/Report/pdf/CommitteeReport.pdf

8. U.S. Department of Health and Human Services. (1996). Physical Activity and Health: A Report of the Surgeon General. Atlanta, GA: U.S. Department of Health and Human Services, Centers for Disease Control and Prevention, National Center for Chronic Disease Prevention and Health Promotion.

9. Pescatello LS, Franklin BA, Fagard R, Farquhar WB, Kelley GA, Ray CA. American College of Sports Medicine position stand. Exercise and hypertension. Med Sci Sports Exerc. 2004; 36(3):533-53.

10. Williams MA, Haskell WL, Ades PA, Amsterdam EA, Bittner V, Franklin BA, et al. Resistance exercise in individuals with and without cardiovascular disease: 2007 update: a scientific statement from the American Heart Association Council on Clinical Cardiology and Council on Nutrition, Physical Activity, and Metabolism. Circulation. 2007; 116(5):572-84.

11. Leitzmann MF, Park Y, Blair A, Ballard-Barbash R, Mouw T, Hollenbeck AR, et al. Physical activity recommendations and decreased risk of mortality. Arch Intern Med. 2007; 167(22):2453-60.

12. Institute for Clinical Systems Improvement (ICSI). Hypertension diagnosis and treatment. Bloomington (US): Institute for Clinical Systems Improvement (ICSI); c2010 [cited 25 May 2011]. Available from: http://www.icsi.org/hypertension_4/hypertension_diagnosis_and_treatment_4.html

13. Colberg SR, Sigal RJ, Fernhall B, Regensteiner JG, Blissmer BJ, Rubin RR, et al. Exercise and type 2 diabetes: the American College of Sports Medicine and the American Diabetes Association: joint position statement. Diabetes Care. 2010; 33(12):e147-67.

14. Mancia G, De Backer G, Dominiczak A, Cifkova R, Fagard R, Germano G, et al. 2007 Guidelines for the management of arterial hypertension: The Task Force for the Management of Arterial Hypertension of the European Society of Hypertension (ESH) and of the European Society of Cardiology (ESC). Eur Heart J. 2007; 28(12):1462-536.

15. Chobanian AV, Bakris GL, Black HR, Cushman WC, Green LA, Izzo JL, Jr., et al. The Seventh Report of the Joint National Committee on Prevention, Detection, Evaluation, and Treatment of High Blood Pressure: the JNC 7 report. JAMA. 2003;289(19):2560-72.

16. Thompson PD, Buchner D, Pina IL, Balady GJ, Williams MA, Marcus BH, et al. Exercise and physical activity in the prevention and treatment of atherosclerotic cardiovascular disease: a statement from the Council on Clinical Cardiology (Subcommittee on Exercise, Rehabilitation, and Prevention) and the Council on Nutrition, Physical Activity, and Metabolism (Subcommittee on Physical Activity). Circulation. 2003;107(24):3109-16.

17. Fagard RH, Bjornstad HH, Borjesson M, Carre F, Deligiannis A, Vanhees L. ESC Study Group of Sports Cardiology recommendations for participation in leisure-time physical activities and competitive sports for patients with hypertension. Eur J Cardiovasc Prev Rehabil. 2005;12(4):326-31.

Treatment algorithm

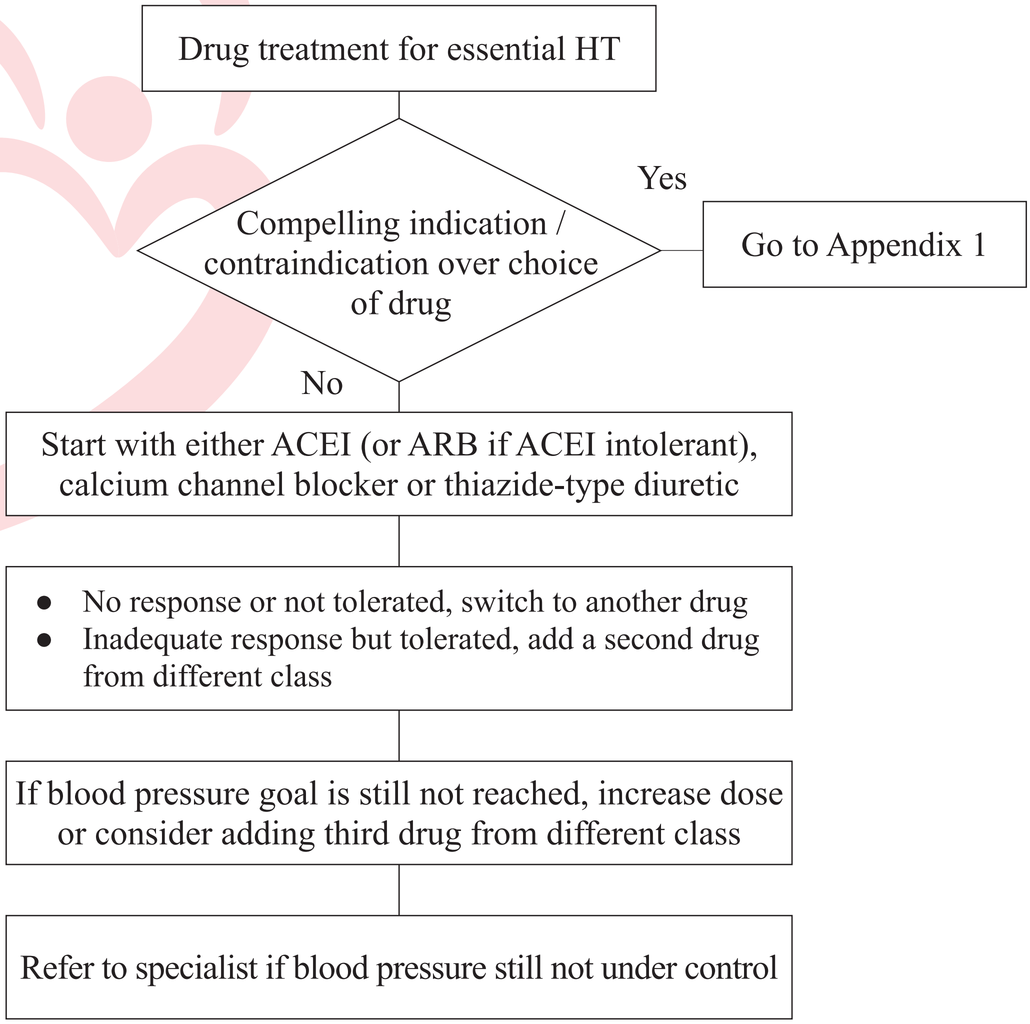

Choices of antihypertensive drugs and goals of therapy

The ultimate goal of anti-hypertensive therapy is to reduce cardiovascular morbidity and mortality. There are excellent clinical outcome trial data proving that lowering blood pressure with different classes of anti-hypertensives, including angiotensin converting enzyme inhibitors (ACEIs), angiotensin receptor blockers (ARBs), beta-blockers (BBs), calcium channel blockers (CCBs), and thiazide-type diuretics, will all reduce the complications of hypertension.[4,6-11,14] It should be emphasized that the perceived risk reduction was directly proportional to blood pressure reduction rather than the drug class used to achieve it,[1] although different drug classes were recognized to have unique benefits specific to individual patient populations.[28]

It has also shown that the lower the blood pressure the better, and that this should be the primary objective of any treatment strategy.[1] Therefore it is important to treat in order to reach the target blood pressure levels whichever drug(s) is/are used[1], i.e. treating SBP and DBP to targets <140/90mmHg for uncomplicated hypertensive patients. In patients with co-morbidities of diabetes or renal disease, the blood pressure target is <130/80mmHg. While for patients with coronary artery disease, the blood pressure should be lowered slowly, and caution is advised in inducing falls of diastolic blood pressure below 60 mmHg if the patient also has diabetes mellitus or is over the age of 60 years.[25]

Within the array of available agents, the choice of drugs will be influenced by many factors, including:

1. The previous, favourable or unfavourable, experience of the individual patient with a given class of drugs.
2. The cost of drugs, either to the individual patient or to the health care provider, although cost considerations should not predominate over efficacy and tolerability in any individual patient.
3. The presence of target organ damage, renal disease, diabetes or the presence of other coexisting cardiovascular disease that may either favour or limit the use of particular classes of antihypertensive drugs. [25] Approach to patients with compelling indications is described in Appendix 1.
4. A significant number of patients require two or more anti-hypertensive drugs in order to achieve blood pressure control.[2,3] The possibility of interactions with drugs used for other conditions present in the patient.
5. There is substantial inter-individual variation in response to single drugs with large absolute falls in some patients, contrasting with little or no response in others.[26]

The family doctor should tailor the choice of drugs to the individual patient, after taking all these factors into account.

With reference to the UK and US guidelines,[5,12] the rationales on selecting different classes of drugs for uncomplicated hypertensive patients are introduced below.

Thiazide-type diuretics

Thiazide-type diuretics have been widely studied and shown to be the drug most likely to confer benefit as first-line treatment for most patients.[11,27,29] In these trials, including ALLHAT[7] thiazide-type diuretics had significantly prevent the cardiovascular complications of hypertension. Thiazide-type diuretics are generally well tolerated and have good blood pressure lowing effect in particular older patients. Thiazide-type diuretics are also more affordable than other antihypertensive agents and are recommended as the initial therapy for most patients with hypertension by US guideline.[5]

Angiotensin-converting enzyme inhibitors (ACEI)/ Angiotensin II Receptor Antagonists (ARB)

It was demonstrated that younger patients and Caucasians tend to have higher serum renin levels than those older patients and the black population, and thus should have better response to ACEI/ ARB which inhibit the renin-angiotensin system.[12,16,17,18] ACEI/ ARB is recommended by the UK guideline[12] as the initial drug choice for people aged <55. Apart from younger patients, ACEI/ ARB are also the drug of choice for patients with heart failure, left ventricular dysfunction, myocardial infarction, ischemic heart disease, diabetic nephropathy, microalbuminuria or history of stroke.

Calcium Channel Blockers (CCB)

Data showed that the blood-pressure lowering effect of CCB is good in most patients. It is well tolerated in general. Clinical trial data also proved that lowering BP with CCB reduces the complications of hypertension.[13,14] It is the most commonly used anti-hypertensive medication by private doctors in Hong Kong.[15]

Beta-blocker

The decision not to recommend Beta-blockers for first line therapy is based on evidence from head-to-head trials[19,20,21,22] that beta-blockers were less effective than the comparator drug at reducing major cardiovascular events, in particular stroke. An additional concern is the increased risk of developing diabetes, particular with the combination of thiazide-type diuretic. However, beta-blockers may be considered in younger people, particularly[24]:

- for those with an intolerance or contraindication to ACE inhibitors and angiotensin-II antagonists or
- for women of child-bearing potential or
- for patients who have previously had a heart attack, angina, heart failure or an irregular heart beat

Appendix 1 Compelling and Possible Indications and Contraindications for the Major Classes of Antihypertensive Drugs[23]

Class of Drug	Compelling Indications	Possible Indications	Compelling Contraindications	Possible Contraindications
ACE Inhibitors (ACEI)	Heart failure, Left ventricular dysfunction, Post myocardial infarction, Diabetic nephropathy	Proteinuric renal disease	Pregnancy, Bilateral renal artery stenosis, Hyperkalaemia	Renal impairment
Angiotensin II Receptor Blockers (ARB)	ACE inhibitor intolerance		Pregnancy, Bilateral renal artery stenosis, Hyperkalaemia	Renal impairment
Alpha-Blockers	Benign prostatic hypertrophy			Orthostatic hypotension
Beta-Blockers	Angina, Post myocardial infarction Tachyarrhythmias	Heart failure (low dose)	Asthma, chronic obstructive pulmonary disease, Heart block	Peripheral vascular disease
Calcium Channel Blockers (dihydropyridine)	Elderly patients, Isolated systolic hypertension	Angina, Peripheral vascular disease		Congestive heart failure
Calcium Channel Blockers (rate limiting, e.g. verapamil, diltiazem)	Angina		Heart block	Congestive heart failure, combination with beta-blockers
Thiazide/ thiazide-like Diuretics	Heart failure, Elderly patients, Isolated systolic hypertension		Gout	Dyslipidaemia, Pregnancy, Sexually active males

Reference:

1. Turnbull F, Blood Pressure Lowering Treatment Trialists' Collaboration. Effects of different blood-pressure-lowering regimens on major cardiovascular events: results of prospectively-designed overviews of randomised trials. Lancet. 2003; 362(9395):1527-35.

2. Cushman WC, Ford CE, Cutler JA, Margolis KL, Davis BR, Grimm RH, et al. Success and predictors of blood pressure control in diverse North American settings: The Antihypertensive and Lipid-Lowering Treatment to Prevent Heart Attack Trial (ALLHAT). J Clin Hypertens (Greenwich). 2002;4(6):393-404.

3. Black HR, Elliott WJ, Neaton JD, Grandits G, Grambsch P, Grimm RH, Jr., et al. Baseline Characteristics and Early Blood Pressure Control in the CONVINCE Trial. Hypertension. 2001; 37(1):12-8.

4. Neal B, MacMahon S, Chapman N. Effects of ACE inhibitors, calcium antagonists, and other blood-pressure-lowering drugs: results of prospectively designed overviews of randomised trials. Blood Pressure Lowering Treatment Trialists' Collaboration. Lancet. 2000;356(9246):1955-64.

5. U.S. Department of Health and Human Services. The seventh report of the Joint National Committee on Prevention, Detection, Evaluation, and Treatment of High Blood Pressure. [Internet]. Bethesda, MD: U.S. Department of Health and Human Services; 2004. Available from: http://www.nhlbi.nih.gov/guidelines/hypertension/jnc7full.pdf

6. Dahlof B, Devereux RB, Kjeldsen SE, Julius S, Beevers G, de Faire U, et al. Cardiovascular morbidity and mortality in the Losartan Intervention For Endpoint reduction in hypertension study (LIFE): a randomised trial against atenolol. Lancet. 2002;359(9311):995-1003.

7. Major outcomes in high-risk hypertensive patients randomized to angiotensin-converting enzyme inhibitor or calcium channel blocker vs diuretic: The Antihypertensive and Lipid-Lowering Treatment to Prevent Heart Attack Trial (ALLHAT). JAMA. 2002;288(23):2981-97.

8. Yusuf S, Sleight P, Pogue J, Bosch J, Davies R, Dagenais G. Effects of an angiotensin-converting-enzyme inhibitor, ramipril, on cardiovascular events in high-risk patients. The Heart Outcomes Prevention Evaluation Study Investigators. N Engl J Med. 2000; 342(3):145-53.

9. PROGRESS Collaborative Group. Randomised trial of a perindopril-based blood-pressure lowering regimen among 6,105 individuals with previous stroke or transient ischaemic attack. Lancet. 2001;358(9287):1033-41.

10. Wing LM, Reid CM, Ryan P, Beilin LJ, Brown MA, Jennings GL, et al. A comparison of outcomes with angiotensin-converting--enzyme inhibitors and diuretics for hypertension in the elderly. N Engl J Med. 2003; 348(7):583-92.

11. Psaty BM, Smith NL, Siscovick DS, Koepsell TD, Weiss NS, Heckbert SR, et al. Health outcomes associated with antihypertensive therapies used as first-line agents. A systematic review and meta-analysis. JAMA. 1997;277(9):739-45.

12. National Collaborating Centre for Chronic Conditions. Hypertension: management in adults in primary care: pharmacological update. London: Royal College of Physicians; 2006. Available from: http://www.nice.org.uk/nicemedia/live/10986/30111/30111.pdf

13. Neal B, MacMahon S, Chapman N. Effects of ACE inhibitors, calcium antagonists, and other blood-pressure-lowering drugs: results of prospectively designed overviews of randomised trials. Blood Pressure Lowering Treatment Trialists' Collaboration. Lancet. 2000; 356(9246):1955-64.

14. Black HR, Elliott WJ, Grandits G, Grambsch P, Lucente T, White WB, et al. Principal results of the Controlled Onset Verapamil Investigation of Cardiovascular End Points (CONVINCE) trial. JAMA. 2003;289(16):2073-82.

15. Chan WK, Chung TS, Lau BS, Law HT, Yeung AK, Wong CH. Management of hypertension by private doctors in Hong Kong. Hong Kong Med J. 2006;12(2):115-8.

16. Sagnella GA. Why is plasma renin activity lower in populations of African origin? J Hum Hypertens. 2001;15(1):17-25.

17. Gibbs CR, Beevers DG, Lip GY. The management of hypertensive disease in black patients. QJM. 1999;92(4):187-92.

18. He FJ, Markandu ND, Sagnella GA, MacGregor GA. Importance of the renin system in determining blood pressure fall with salt restriction in black and white hypertensives. Hypertension. 1998;32(5):820-4.

19. MRC trial of treatment of mild hypertension: principal results. Medical Research Council Working Party. Br Med J (Clin Res Ed). 1985;291(6488):97-104.

20. Medical Research Council trial of treatment of hypertension in older adults: principal results. MRC Working Party. BMJ. 1992;304(6824):405-12.

21. Dahlof B, Sever PS, Poulter NR, Wedel H, Beevers DG, Caulfield M, et al. Prevention of cardiovascular events with an antihypertensive regimen of amlodipine adding perindopril as required versus atenolol adding bendroflumethiazide as required, in the Anglo-Scandinavian Cardiac Outcomes Trial-Blood Pressure Lowering Arm (ASCOT-BPLA): a multicentre randomised controlled trial. Lancet. 2005;366(9489):895-906.

22. Zanchetti A, Bond MG, Hennig M, Neiss A, Mancia G, Dal Palu C, et al. Calcium antagonist lacidipine slows down progression of asymptomatic carotid atherosclerosis: principal results of the European Lacidipine Study on Atherosclerosis (ELSA), a randomized, double-blind, long-term trial. Circulation. 2002;106(19):2422-7.

23. Williams B, Poulter NR, Brown MJ, Davis M, McInnes GT, Potter JF, et al. Guidelines for management of hypertension: report of the fourth working party of the British Hypertension Society, 2004-BHS IV. J Hum Hypertens. 2004;18(3):139-85.

24. National Collaborating Centre for Chronic Conditions. Hypertension: management in adults in primary care: pharmacological update. London: Royal College of Physicians; 2006. Available from: http://www.nice.org.uk/nicemedia/live/10986/30111/30111.pdf.

25. Rosendorff C, Black HR, Cannon CP, Gersh BJ, Gore J, Izzo JL, Jr., et al. Treatment of hypertension in the prevention and management of ischemic heart disease: a scientific statement from the American Heart Association Council for High Blood Pressure Research and the Councils on Clinical Cardiology and Epidemiology and Prevention. Circulation. 2007;115(21):2761-88.

26. Attwood S, Bird R, Burch K, Casadei B, Coats A, Conway J, et al. Within-patient correlation between the antihypertensive effects of atenolol, lisinopril and nifedipine. J Hypertens. 1994;12(9):1053-60.

27. Psaty BM, Lumley T, Furberg CD, Schellenbaum G, Pahor M, Alderman MH, et al. Health outcomes associated with various antihypertensive therapies used as first-line agents: a network meta-analysis. JAMA. 2003;289(19):2534-44.

28. Law MR, Morris JK, Wald NJ. Use of blood pressure lowering drugs in the prevention of cardiovascular disease: meta-analysis of 147 randomised trials in the context of expectations from prospective epidemiological studies. BMJ. 2009;338:b1665.

29. Wright JM, Musini VM. First-line drugs for hypertension. Cochrane Database Syst Rev. 2009(3):CD001841.

Module 8 Annual Assessment

The aims of the annual assessment are:

1. to detect cardiovascular, cerebrovascular and renal complications
2. to assess patient's knowledge, attitude and compliance in respect of living with hypertension and drug treatment
3. to give appropriate advice to patient on healthy lifestyle and cardiovascular related risk factors
4. to ensure satisfactory control over the year (average of most recent three readings of <140/90 mmHg)

History:

1. New symptoms of cardiovascular complications like angina, neurological symptoms
2. Checking smoking status
3. Alcohol intake
4. Exercise
5. Family history of premature coronary heart disease
6. Assessing patient's ideas and concerns about hypertension, side effects of drugs, compliance to treatment and effect on quality of life

Physical examination[1,2,3]:

1. Measure blood pressure with a mercury sphygmomanometer or validated electronic device
2. Check body mass index
3. Cardiovascular examination including peripheral pulses, bruits

Laboratory Investigation[1]:

Recommended Tests	*Justifications*
General	
• Fasting blood glucose	• Detection of concomitant diabetes<br>• Guidance for cardiovascular risk factor control
• Urine for protein/ albumin	• Detection of hypertensive nephropathy
• Uric acid if patient is on diuretic	• Detection of diuretic-induced hyperuricaemia
• Optional - Random spot urine albumin: creatinine ratio (ACR)	• Detection of hypertensive nephropathy
Renal function test	
• Sodium, Potassium, Urea, Creatinine	• Detection of hypertensive nephropathy<br>• Detection of diuretic-induced electrolyte disturbance
Lipid profiles	
• Total Cholesterol<br>• Triglyceride<br>• HDL-Cholesterol<br>• LDL-Cholesterol	• Detection of concomitant cardiovascular risk factors<br>• Guidance for antihypertensive and lipid-lowering pharmacotherapy

Management:

1. Review the risk factors and blood results
2. Assess the side effects of drug treatment and manage accordingly
3. Inform and encourage patient on lifestyle modifications like salt reduction and exercise
4. Explore reasons for non-compliance
5. Ensure patient understands nature of hypertension and benefits of long-term therapy and follow-up

Reference:

1. HA Clinical Practice Guideline. Management of Hypertension in Primary Care. Hong Kong SAR: Hospital Authority; 2008.

2. Whitworth JA. 2003 World Health Organization (WHO)/International Society of Hypertension (ISH) statement on management of hypertension. J Hypertens. 2003;21(11):1983-92.

3. Williams B, Poulter NR, Brown MJ, Davis M, McInnes GT, Potter JF, et al. British Hypertension Society guidelines for hypertension management 2004 (BHS-IV): summary. BMJ. 2004;328(7440):634-40.
