# Supplementary material for: The adoption of hypertension reference framework: An investigation among primary care physicians of Hong Kong
Source: PLoS One. 2018 Oct 9;13(10):e0205529. doi: 10.1371/journal.pone.0205529 (PMC6177174; doi:10.1371/journal.pone.0205529)
Supplement: S1 Table — (DOCX) [file pone.0205529.s002.docx]

**S1 Table.** **Potential factors influencing the adoption of the Reference Framework**

| ***Guideline-related enablers*** | Proportion of agree /strongly agree (%) | *p* |
| --- | --- | --- |
| 1. Inclusion of essential clinical information for hypertension management. | 371 (96.9) | 0.343 |
| 1. **Inclusion of sufficient local information.*** | **357 (93.2)** | **0.017*** |
| 1. Presence of multiple versions meeting different needs of primary care physicians. | 312 (81.5) | 0.435 |
| 1. Improving patients’ knowledge on hypertension management. | 352 (91.9) | 0.957 |
| 1. Infrequent update on the information of the framework. | 267 (69.7) | 0.876 |
| 1. High complexity of the framework. | 217 (56.7) | 0.958 |
| 1. Inclusion of sufficient recommendations with high validity. | 352 (91.9) | 0.599 |
| 1. Inclusion of adequate and high quality evidences. | 354 (92.4) | 0.485 |
| ***Guideline-related barriers*** | Proportion of agree /strongly agree (%) | *p* |

| 1. Lack of new recommendations compared with currently existing guidelines. | 237 (61.9) | 0.965 |
| --- | --- | --- |

| 1. Restriction on the choices of medical services. | 210 (54.8) | 0.207 |
| --- | --- | --- |
| 1. Reduction of professional autonomy of primary care physicians. | 194 (50.7) | 0.189 |
| ***Patient-related barriers*** | Proportion of agree /strongly agree (%) | *p* |
| 1. Limited knowledge about Hypertension and its complications. | 306 (79.9) | 0.613 |
| 1. Low motivation to change their lifestyles into the recommended ones. | 330 (86.2) | 0.139 |
| 1. Lack of concern on their own health. | 268 (70.0) | 0.417 |
| 1. Low adherence to therapeutic regimen. | 267 (69.7) | 0.631 |
| 1. Difficulties accessing the electronic patient-version framework. | 221 (57.7) | 0.268 |
| 1. Language barriers on understanding the framework recommendations. | 201 (52.5) | 0.063 |
| ***Primary care physicians-related barriers*** | Proportion of agree /strongly agree (%) | *p* |
| 1. Low awareness of the framework before this survey. | 209 (54.6) | 0.380 |
| 1. Lack of motivation for changing your clinical practice. | 195 (50.9) | 0.426 |
| 1. Lack of expectancy of this framework on improving hypertension care of the patients. | 196 (51.2) | 0.934 |
| 1. Judgment for treatments were made mainly based on individual patients’ health conditions. | 287 (74.9) | 0.801 |
| ***External factors barriers*** | Proportion of agree /strongly agree (%) | *p* |
| 1. Insufficient allied health support. | 206 (53.8) | 0.618 |
| 1. The framework cannot be integrated into your current clinical setting. | 136 (35.5) | 0.486 |
| 1. Hindrance from higher authorities or other stakeholders. | 140 (36.6) | 0.455 |
| 1. Peer pressure. | 115 (30.0) | 0.153 |
| 1. Market competition. | 133 (34.7) | 0.802 |
| 1. Limited consultation time. | 255 (66.6) | 0.748 |
| 1. Limited resources. | 220 (57.4) | 0.825 |

The Chi-square tests were employed for the analysis of categorical data.

*P-value <0.05 is considered as at significant level.
